# ﻿A review of the *Augochloropsis* (Hymenoptera, Halictidae) and keys to the shiny green Halictinae of the midwestern United States

**DOI:** 10.3897/zookeys.1130.86413

**Published:** 2022-11-18

**Authors:** Zachary M. Portman, Mike Arduser, Ian G. Lane, Daniel P. Cariveau

**Affiliations:** 1 Department of Entomology, University of Minnesota, St Paul, MN, USA University of Minnesota St Paul United States of America; 2 Conservation Research Institute, Cedarburg, WI, USA Conservation Research Institute Cedarburg United States of America

**Keywords:** *Agapostemon*, *Augochlora*, *Augochlorella*, identification, *Paraugochloropsis*, species complex

## Abstract

*Augochloropsis* and other shiny green Halictinae have had various taxonomic issues and are often misidentified. One prevailing taxonomic issue is that *Augochloropsismetallica* (Fabricius) has two subspecies, that have long been recognized as morphologically distinct (*Augochloropsismetallicametallica* and *Augochloropsismetallicafulgida* (Smith), but the subspecies are inconsistently applied in the literature. Here, we review the *Augochloropsis* of the Midwest and further address the *Augochloropsis* species in the broader United States to resolve the outstanding taxonomic issues with the midwestern species. We provide identification keys and diagnoses for the genera and species of the shiny green Halictinae of the midwestern United States, which includes the genera *Agapostemon*, *Augochlora*, *Augochlorella*, and *Augochloropsis*. This work results in taxonomic changes to *Augochloropsis*. *Augochloropsissumptuosa* (Smith) is split into two species, with the name *Augochloropsissumptuosa* retained for the eastern form, and *Augochloropsishumeralis* (Patton), **stat. nov.**, reinstated for the western form. *Augochloropsismetallica* is split into five species, with two of those species occurring in the midwestern United States: *Augochloropsismetallica* and *Augochloropsisviridula* (Smith), **stat. nov.** Examination of the holotype of *Augochloropsisfulgida* (Smith) revealed that it does not agree with the prevailing concept of *Augochloropsismetallicafulgida*; it is reinstated as *Augochloropsisfulgida*, **stat. nov.**, but is currently known only from the holotype female from Florida. *Augochloropsiscuprea* (Smith), long considered to be a synonym of *Augochloropsismetallica*, is also distinct, and we are reinstating *Augochloropsiscuprea*, **stat. nov.**, though the range of this species is unclear. We further recognize *Augochloropsisfulvofimbriata* (Friese), **stat. nov.**, from South and Central America, as distinct. These changes result in a total of three *Augochloropsis* species in the Midwest and seven named species in the United States. We are aware of additional species from the southern and southwestern United States that are undescribed, and we highlight additional taxonomic work that remains to be done.

## ﻿Introduction

The genus *Augochloropsis* Cockerell contains approximately 140 species, recognizable by their metallic coloration and distinctly-shaped tegula (Hurd 1979; Engel 2000; Michener 2007; Ascher and Pickering 2022) and occurs throughout most of the Western Hemisphere (Moure and Hurd 1987). *Augochloropsis* was originally erected as a subgenus of *Augochlora* Say by Cockerell (1897) and later elevated to genus by Schrottky (1906). This classification was later confirmed by the in-depth generic revision of augochlorine bees by Eickwort (1969). The number of *Augochloropsis* species that occur in the United States has been a matter of some debate, with different authors variously claiming that there are anywhere from two to five species (Sandhouse 1937 claimed two, Dreisbach 1945 claimed five, though did not list them all). In the most recent treatment, covering the Eastern United States, Mitchell (1960) recognized three species and one additional subspecies.

The species of *Augochloropsis* in the United States and Canada have undergone many taxonomic changes. In the first revisionary work, Sandhouse (1937) recognized two species: *Augochloropsiscaerulea* (Ashmead) and *Augochloropsiscuprea* (Smith). Dreisbach (1945) recognized that what Sandhouse regarded as *Augochloropsiscuprea* was in fact two species in his region and split it into *Augochloropsiscuprea* and *Augochloropsisviridula* (Smith). Dreisbach (1945) also replaced the name *Augochloropsiscaerulea* with the earlier name of *Augochloropsishumeralis* (Patton). After examination of the Fabricius types, and apparently unaware of the work by Dreisbach, Moure (1960) synonymized *Augochloropsiscuprea* with the older name *Augochloropsismetallica* (Fabricius). Moure (1960) also split *Augochloropsismetallica* into subspecies, suggesting *Augochloropsismetallicafulgida* (Smith) for the “southern variety.” Moure’s classification was followed by the most recent treatment performed by Mitchell (1960), who recognized two subspecies of *Augochloropsismetallica*, replaced the name *Augochloropsishumeralis* with *Augochloropsissumptuosa* (Smith) based on correspondence with Moure, and recognized a third species, *Augochloropsisanonyma* Cockerell.

There has been inconsistent use of the names and species concepts of the *Augochloropsis* in the United States in recent bee diversity studies, primarily with the usage of the subspecies of *Augochloropsismetallica*, with some researchers recognizing them and others not. As a result, when papers refer to “*Augochloropsismetallica*” it is often impossible to know whether they were referring to “*Augochloropsismetallicametallica*” or “*Augochloropsismetallicafulgida*,” especially given that many publications fail to cite the identification resources or taxon concepts they used (see Packer et al. 2018). More recently, several authors have recognized four species of *Augochloropsis* in the United States, informally elevating the subspecies *Augochloropsismetallicafulgida* (Smith) to full species (e.g., Camilo et al. 2018; Stephenson et al. 2018; Decker et al. 2020), but this has not been widely adopted. Overall, this has made it difficult to compare different publications and has hindered our ability to build even a basic understanding of the ranges and patterns of occurrence of these two taxa.

More broadly, the green Halictinae in general are plagued by misidentifications, particularly of males. This is in part due to the lack of up-to-date or high-quality identification resources. Indeed, some widely used identification resources contain characters that are too variable to be useful or are outright incorrect (e.g., the keys on discoverlife.org; Ascher and Pickering 2022). To help alleviate this issue, we continue the tradition of regional keys of shiny green Halictinae (e.g., Lovell 1942; Dreisbach 1945). Our keys cover the midwestern United States, defined as containing the states Illinois, Indiana, Iowa, Michigan, Minnesota, Missouri, Ohio, and Wisconsin. We do not include the Dakotas, Nebraska, and Kansas due to the shift from eastern to western fauna that occurs within these states, though the keys will still work in the easternmost parts of these states.

Here, we review the *Augochloropsis* species of the Midwest, recognizing three species from the region: *Augochloropsishumeralis*, *Augochloropsismetallica* sensu stricto, and *Augochloropsisviridula*. While we originally aimed to simply clarify the subspecies of *Augochloropsismetallica*, it necessarily expanded into a larger project after examination of the type specimens revealed numerous issues that necessitated a geographic expansion and a more in-depth update of the taxonomy. As a result of the updated taxonomy, we are making the following changes: *Augochloropsishumeralis* is resurrected from synonymy with *Augochloropsissumptuosa*, we define *Augochloropsisfulgida* in a different sense than it has traditionally been used, and *Augochloropsisviridula* is resurrected from synonymy and recognized as a valid species. We further recognize as valid species two former synonyms of *Augochloropsismetallica: Augochloropsiscuprea* and *Augochloropsisfulvofimbriata* Friese. We also point to more work that remains to be done, as we recognize seven species in the United States, but there appear to be at least four more undescribed or unrecognized species. Lastly, we provide an illustrated key to the *Augochloropsis* and the other shiny green Halictinae of the midwestern United States, which covers the genera *Agapostemon* Guérin-Méneville, *Augochlora*, *Augochlorella* Sandhouse, and *Augochloropsis*.

## ﻿Materials and methods

The keys used here are variously adapted and modified from existing sources, primarily from Arduser (2015) and Mitchell (1960), but also incorporate pieces and characters from Sandhouse (1937), Lovell (1942), Dreisbach (1945), Ordway (1966), and Michener et al. (1994). Various novel characters are also included. Higher-level classification and morphological terminology follow Michener (2007), with “metasoma” used for what is colloquially called the abdomen, and metasomal tergum and sternum are abbreviated to **T** and **S**, respectively. Antennal flagellomeres are abbreviated to **F**.

The keys and diagnoses follow the species concepts from the most recent revisions of those groups:

- *Agapostemon*: Roberts (1972).
- *Augochlorella*: Coelho (2004).
- *Augochlora*: Mitchell (1960).
- *Augochloropsis*: taxon concepts revised here.

The following museum and collection acronyms are used in the paper:

**ANSP**The Academy of Natural Sciences of Drexel University, Philadelphia, Pennsylvania, USA (J. Weintraub).

**NHMUK**The Natural History Museum, London, United Kingdom (J. Monks).

**CNBL** The collection of the Cariveau Native Bee Lab, St. Paul Minnesota, USA (Z. Portman).

**CRC** Catherine Reed Collection. Currently resides in the Cariveau Native Bee Lab and will be accessioned into the UMSP.

**EERC** Elaine Evans Research Collection (E. Evans). Housed at the Cariveau Native Bee Lab (CNBL) and will be accessioned into the UMSP.

**IDNP** Indiana Dunes National Park. Examined specimens deposited at the UMSP.

**iNat** Selected high-quality records from the community science portal iNaturalist.com were examined for *Augochloropsishumeralis*. All record information is included in the material examined section.

**MASR** Mike Arduser specimen record. Includes a combination of specimens in Mike Arduser’s personal collection, as well as specimens Mike Arduser has personally identified but no longer has in hand.

**MNDNR** The Minnesota Department of Natural Resources, St. Paul, MN, USA (J. Petersen and N. Gerjets). These are primarily deposited in the UMSP except for a small synoptic collection.

**NHMD** Natural History Museum of Denmark, Copenhagen, Denmark (L. Vilhelmsen).

**USNM**Smithsonian National Museum of Natural History, Washington D.C., USA.

**OSUC** C.A. Triplehorn Insect Collection, Ohio State University Columbus, Ohio, USA (L. Musetti).

**OUMNH** University Museum of Natural History, Oxford, United Kingdom (J. Hogan).

**UMSP**University of Minnesota Insect Collection, St. Paul, Minnesota, USA (R. Thomson).

This study represents material from multiple sources, often examined over the course of many years. As a result, in the reports of material examined we are reporting a combination of specimen-level and county-level data. Historic specimens were manually georeferenced using Google Earth Pro software (v. 7.3.4.8248). For county level records, points were mapped to the county centroids. Specimen images were taken using an Olympus DP27 camera mounted on an Olympus SZX16 stereo microscope, with the images stacked using CombineZP software (Hadley 2010). Images of type specimens were provided by the type depositories. Figures were made with Adobe Photoshop software. Maps were created using the R statistical environment (R Core Team 2022), using both the ‘*ggplot2*’ package (Wickham 2016) and the ‘*sf*’ package (Pebesma 2018). State and province borders were imported from the ‘*rnaturalearth*’ package (South 2017).

## ﻿Results and systematics

Identification of the shiny green Halictinae of the midwestern United States.

### ﻿Key to genera

**Table:** 

1	Tegula enlarged and asymmetric, with the inner posterior margin hooked or angled (Fig. 1A)	***Augochloropsis* Cockerell**
–	Tegula normal and ovoid (Fig. 1B)	**2**
2	Propodeum with posterior surface encircled by a raised rim or carina (Fig. 2A, B); males with black and yellow striped metasoma	***Agapostemon* Guérin-Méneville**
–	Propodeum with posterior surface not encircled by a carina (Fig. 2C, D), though lateral carinae may be present; males with metasoma metallic green	**3**
3	Female S1 with central keel (Fig. 3A); both sexes with paraocular lobe prominent, forming a rounded acute angle (Fig. 3C, E); female with apex of mandible with two large and equal-sized teeth; male with posterior and lateral faces of propodeum closely, distinctly punctate (Fig. 3B) and S4 apical margin entire	***Augochlorapura* (Say)**
–	Female S1 without keel; both sexes with paraocular lobe not prominent, forming an obtuse or right angle (Fig. 3D, F); mandible with a small preapical tooth; male with posterior and lateral faces of propodeum rugose to rugosopunctate, punctures obscure (Fig. 2D), and S4 apical margin weakly to strongly concave (compare to S3, which is entire)	***Augochlorella* Sandhouse**

**Figure 1.**
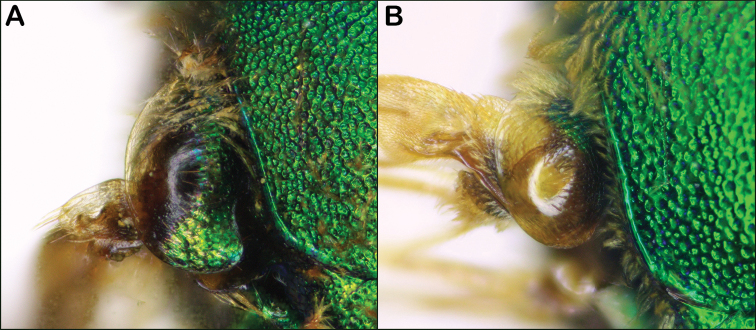
**A** tegula of *Augochloropsis*, enlarged and asymmetric, with the inner posterior margin angled (*Augochloropsismetallica* female pictured) **B** normal oval tegula (*Agapostemonsplendens* female pictured).

#### ﻿Genus Agapostemon

Taxon classificationAnimaliaHymenopteraHalictidae

Guérin-Méneville

8D7C203F-8557-5AD3-B9B4-F4D1076EF99D

##### Diagnosis.

**Figure 2.**
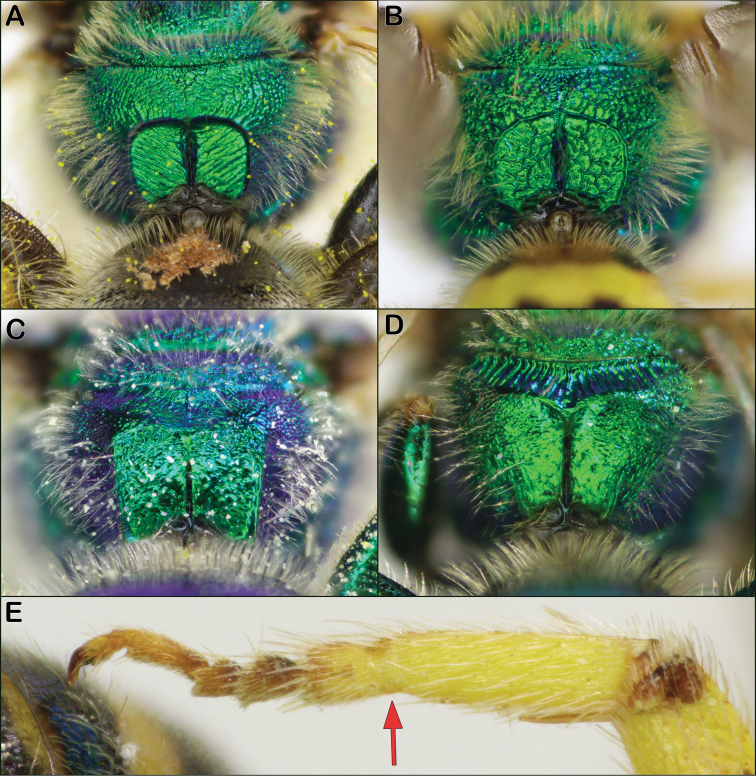
Characters to separate *Agapostemon* from Augochlorini: Complete raised carina on the rear face of the propodeum in *Agapostemon***A** female and **B** male. Incomplete carina in **C***Augochloropsis* female and **D***Augochlorella* male **E***Agapostemon* male hind leg with first two tarsomeres fused (red arrow pointing to point of fusion).

Both sexes of *Agapostemon* are diagnosed by the complete carina on the rear face of the propodeum (Fig. 2A, B). Other metallic green Halictinae genera, such as *Augochloropsis*, can have a pair of lateral carinae (e.g., Fig. 2C), but these are well-separated dorsally and never forming a complete carina as in *Agapostemon*. Females can be further recognized by having the hind tibial spurs with broad teeth. Males can be further recognized by having the metasoma black and yellow striped rather than metallic green and by having the basitarsus fused with the next tarsal segment (Fig. 2E).

**Figure 3.**
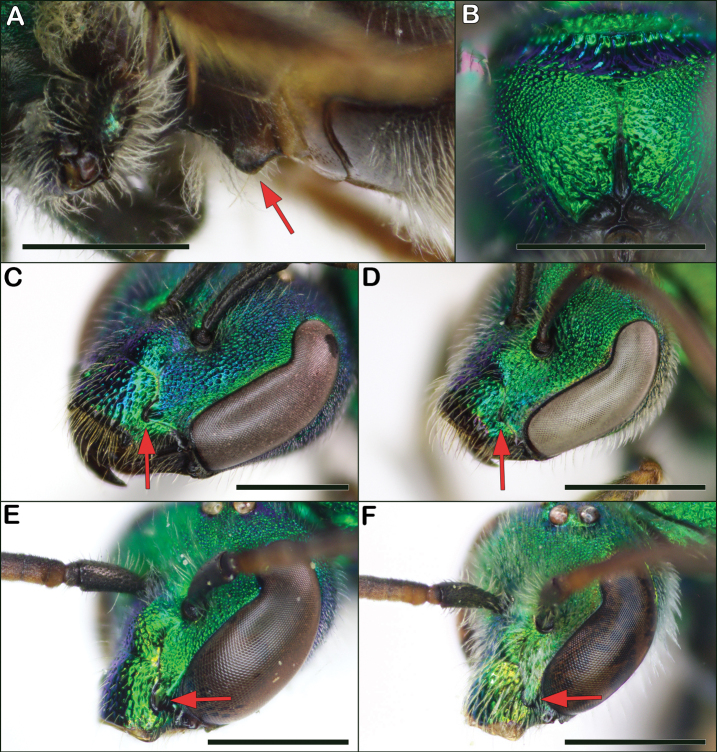
Characters to separate *Augochlora* and *Augochlorella***A** S1 of *Augochlorapura* female with a median keel indicated by red arrow **B***Augochlorapura* male rear propodeum showing distinct punctures **C***Augochlorapura* female face with protruding paraocular lobe indicated by red arrow **D***Augochlorellaaurata* female face with undeveloped paraocular lobe indicated by red arrow **E***Augochlorapura* male face with protruding paraocular lobe indicated by red arrow **F***Augochlorellaaurata* male with undeveloped paraocular lobe indicated by red arrow. Scale bars: 1 mm.

### ﻿Keys to the midwestern species of *Agapostemon*

**Note**: We include two principally western species, *Agapostemonangelicus* Cockerell and *Agapostemonmelliventris* Cresson, that may potentially occur in the midwestern states.

**Key to females**

**Table:** 

1	Metasoma black or amber-colored (Fig. 4A, B)	**2**
–	Metasoma metallic green like thorax (Fig. 4C)	**3**
2	Metasomal terga blackish (Fig. 4A), and clypeus blackish apically; common and widespread species	***virescens* (Fabricius)**
–	Metasomal terga amber-colored, at least in part (Fig. 4B); clypeus yellow apically; Great Plains species in part, furthest eastern occurrence in eastern Kansas	***melliventris* Cresson**
3	Scutum and scutellum “doubly punctate”, i.e., with uniformly scattered large punctures among the more numerous small ones (Fig. 5A, note that this is a variable character with quite a bit of variation in the size and density of the punctures)	***texanus* Cresson or *angelicus* Cockerell**
–	Scutum densely punctate, rugosopunctate or weakly reticulate (Fig. 5B, C), but not “doubly punctate”	**4**
4	Pronotum with dorsolateral angle pointed and produced at a distinct right angle; dorsolateral ridge sharply edged (Fig. 5B, red outline); scutum more reticulate (Fig. 6A); ventral pleural tubercle flush with rest of plate (Fig. 6C)	***sericeus* (Forster)**
–	Pronotum with dorsolateral angle and dorso-lateral ridge blunted (Fig. 5C, red outline), not pointed or sharply edged; scutum with more distinct punctures (Fig. 6B); ventral pleural tubercle upraised, not flush with rest of plate (Fig. 6D)	***splendens* (Lepeletier)**

**Figure 4.**
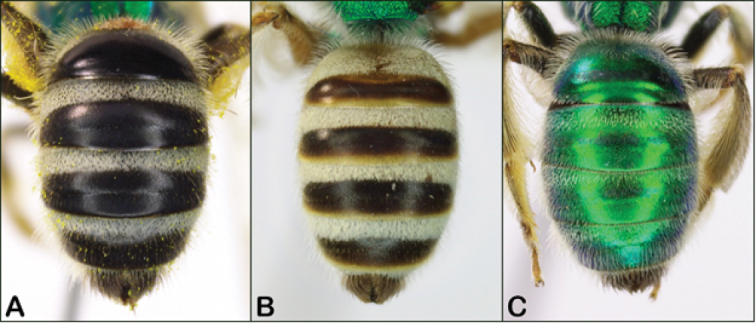
*Agapostemon* female metasomas **A** entirely black (*Agapostemonvirescens* pictured) **B** dark brown with amber (*Agapostemonmelliventris* pictured), note this is a darker specimen **C** metallic green (*Agapostemontexanus* pictured).

**Figure 5.**
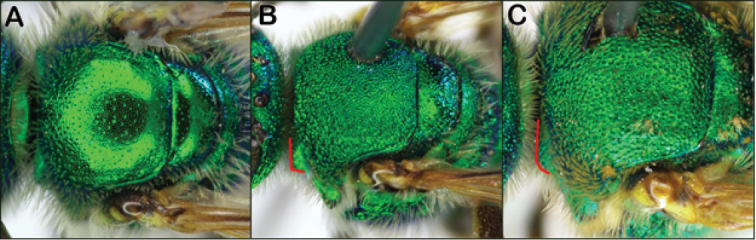
*Agapostemon* female pronotal collar and scutum **A***Agapostemontexanus* doubly punctate **B***Agapostemonsericeus* rugosopunctate with sharp pronotal angle outlined in red **C***Agapostemonsplendens* densely punctate with obtuse pronotal angle outlined in red.

**Figure 6.**
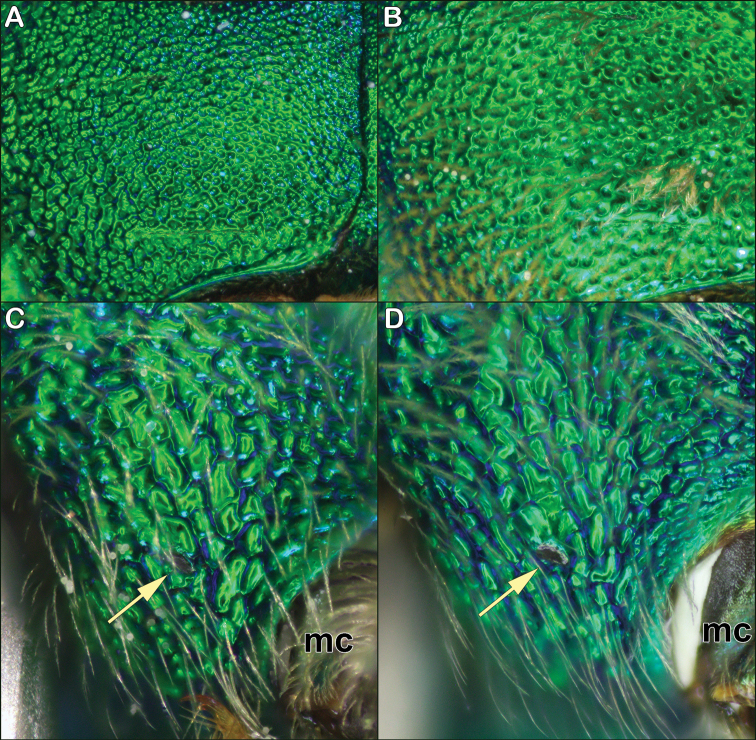
*Agapostemon* thorax characters (anterior of bee to left, “mc” refers to the base of the middle coxa) **A***Agapostemonsericeus* close-up of rugosopunctate scutum **B***Agapostemonsplendens* close-up of densely punctate scutum **C***Agapostemonsericeus* with ventral pleural tubercle flush with rest of plate **D***Agapostemonsplendens* with ventral pleural tubercle slightly upraised. Yellow arrows indicate the ventral pleural tubercle.

**Key to males**

**Table:** 

1	S4 entirely flat, without a low, transverse swelling along the apical or preapical margin (Fig. 7A); S5 and S6 usually all dark brown to black, without any yellow maculations	***virescens* (Fabricius)**
–	S4 (and S3 to a lesser extent) with a low, transverse swelling along the apical or preapical margin (Fig. 7B–D; best seen in oblique lateral view); S5 and S6 usually (but not always) yellow in part (e.g., Fig. 7B)	**2**
2	Hind femur skinny, not swollen at all (Fig. 8A); metasoma mostly yellow (Fig. 9A); Great Plains species in part, furthest eastern occurrence in eastern Kansas	***melliventris* Cresson**
–	Hind femur moderately to grossly swollen (Fig. 6B–D); metasoma with large black bands (e.g., Fig. 9B)	**3**
3	Hind legs quite swollen, width of hind femur about half the length (Fig. 8C)	***splendens* (Lepeletier)**
–	Hind legs only moderately swollen (Fig. 8B, D)	**4**
4	F1 slightly more than half length of F2 (Fig. 10A, antennae should be viewed on the lighter portion where it meets the brown portion); wings slightly brownish	***sericeus* (Forster)**
–	F1 at least three-fourths length of F2 (Fig. 10B); wings clear	**5**
5	Hind tibia with brown to black stripe present anteriorly (and posteriorly) (e.g., Fig. 8D for anterior view), or, if lacking anterior stripe, then also without black stripe on posterior surface; genitalia with relatively large medial plate, base of apical stylus of gonostylus not inflated (Fig. 12); widespread across North America	***texanus* Cresson**
–	Lacking brown to black stripe on anterior surface of hind tibia (Fig. 11A), but stripe present on posterior surface (Fig. 11B); genitalia with small medial plate, basal stylus slightly inflated (Fig. 12); primarily western species	***angelicus* Cockerell**

**Figure 7.**
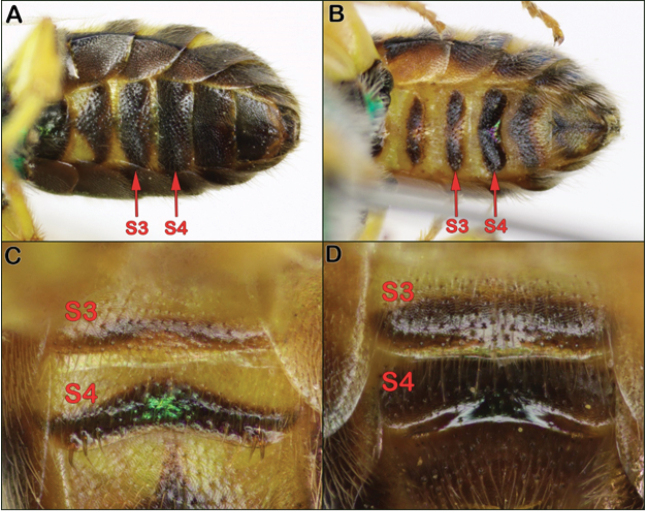
**A***Agapostemonvirescens* metasomal sterna with unmodified S3 and S4 **B***Agapostemontexanus* metasomal sterna with swelling on S4 (and S3 to a lesser extent) **C***Agapostemonsericeus* modified S3 and S4 **D***Agapostemontexanus* modified S3 and S4.

**Figure 8.**
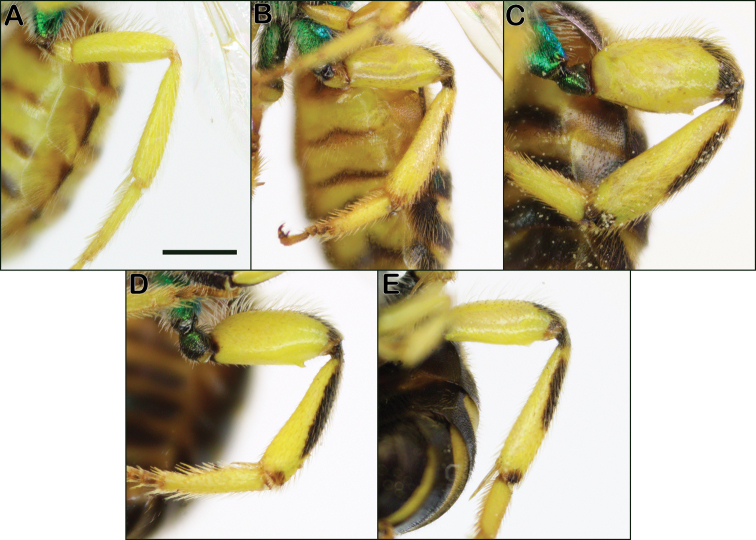
Male *Agapostemon* hind legs **A***Agapostemonmelliventris***B***Agapostemonsericeus***C***Agapostemonsplendens***D***Agapostemontexanus***E***Agapostemonvirescens*. Scale bar 1 mm, all images at the same scale.

**Figure 9.**
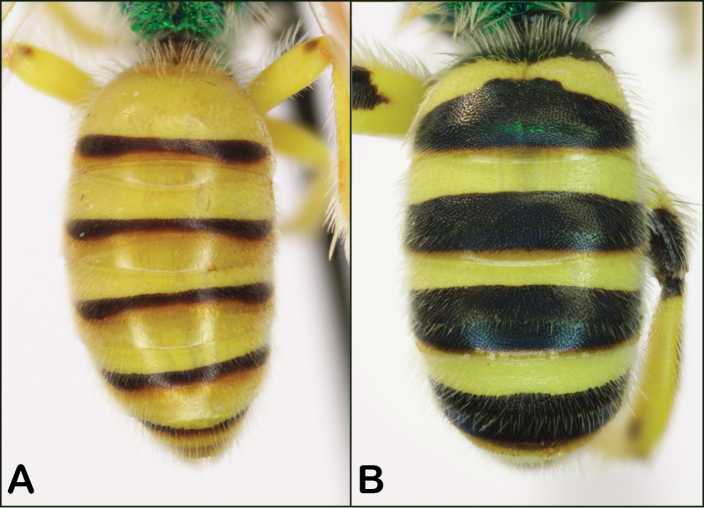
Male metasomal coloration **A***Agapostemonmelliventris* with mostly yellow metasoma **B***Agapostemontexanus* with mostly dark metasoma.

**Figure 10.**
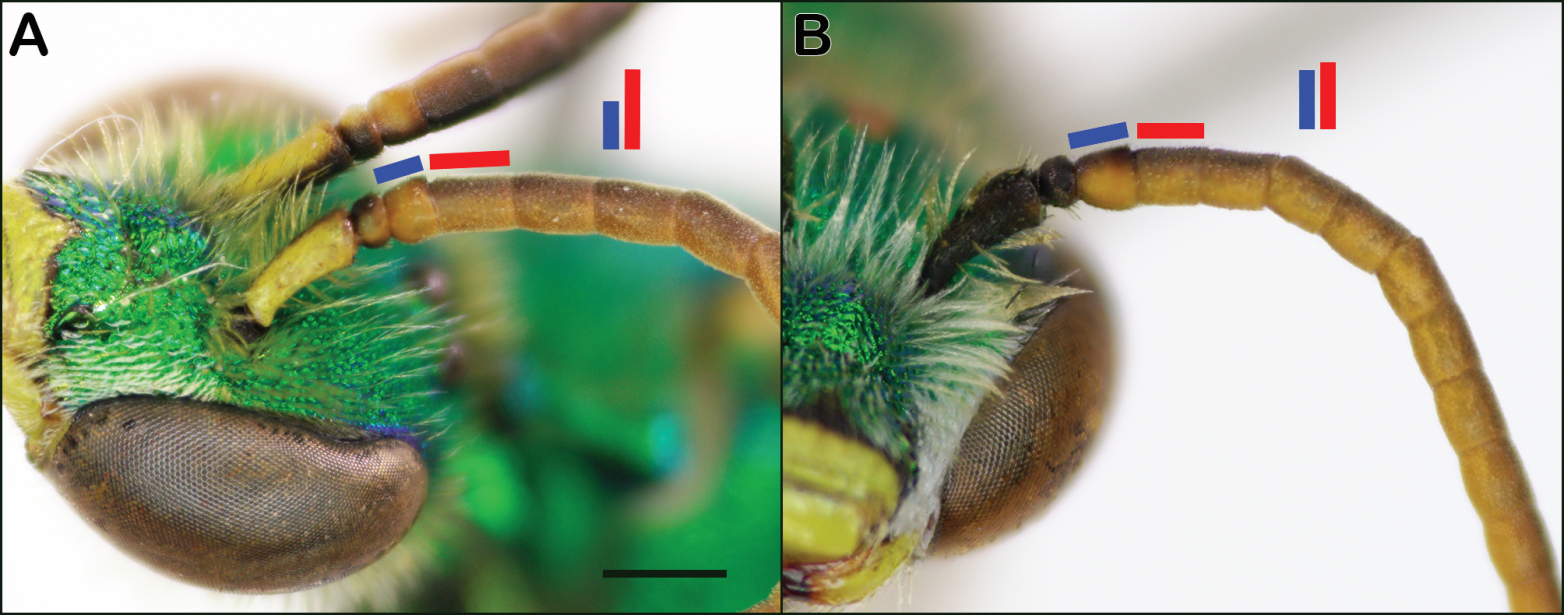
Male antennae with segments highlighted with bars to help illustrate their relative lengths **A***Agapostemonsericeus***B***Agapostemontexanus*. Scale bars: 500 µm, both images at same scale.

**Figure 11.**
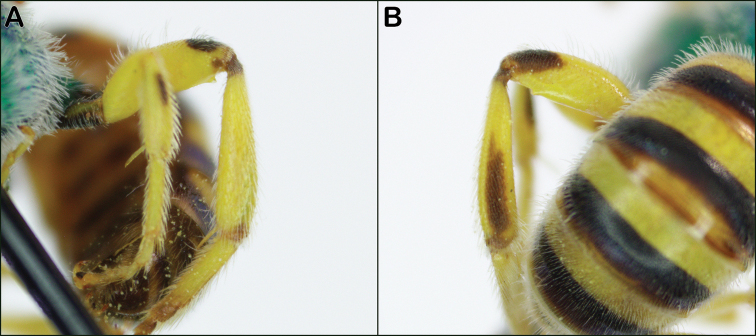
*Agapostemonangelicus* hind tibia **A** anterior view of tibia lacking a dark mark **B** posterior view of tibia with dark mark present.

**Figure 12.**
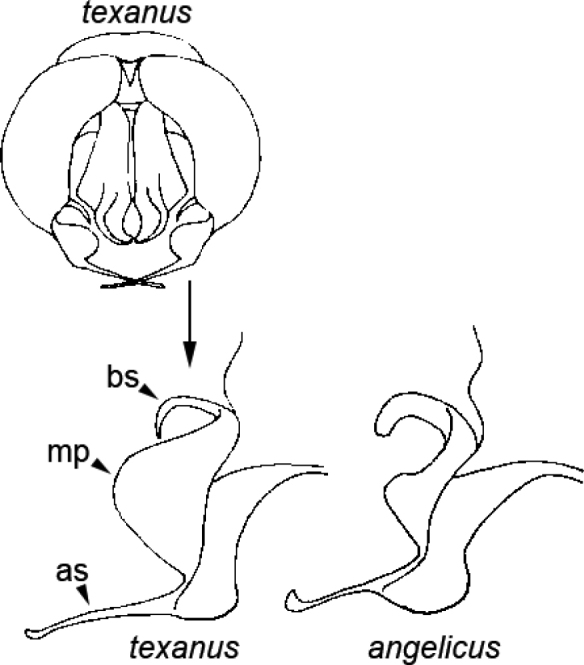
*Agapostemontexanus* and *Agapostemonangelicus* genitalia. Abbreviations: bs = basal stylus, mp = medial plate, as = apical stylus. Figure modified from Roberts (1972).

#### ﻿ Agapostemon (Agapostemon) angelicus

Taxon classificationAnimaliaHymenopteraHalictidae

Cockerell

CB0F60EB-D0D2-55B1-8B78-34D95C5301EE

##### Diagnosis.

Females of *Agapostemonangelicus* can be recognized by the unique double-punctate scutum (as in Fig. 5A), a character they share with *Agapostemontexanus*. Our efforts to find characters to separate the females of these two species for the most part have been fruitless, and like workers before us (Roberts 1972), we consider females of *Agapostemonangelicus* and *Agapostemontexanus* to be morphologically indistinguishable. Males of *Agapostemonangelicus* can be separated from males of *Agapostemontexanus* using the leg and genitalia characters given in the key.

##### Remarks.

*Agapostemonangelicus* is primarily a western species, though Roberts (1972) records it from central Iowa and extreme eastern Kansas. As far as we have been able to determine, there are no recent records of *Agapostemonangelicus* east of the 98^th^ Meridian. A 2018–2019 statewide survey of Kansas bees by Morphew (2017) did not find any *Agapostemonangelicus* males (verified by MSA) east of the central part of the state, with the easternmost records from Ellsworth and Rice counties. Recent survey efforts in eastern Nebraska have not found any *Agapostemonangelicus* east of the Grand Island area (Hall Co.). No confirmed recent records are known from Minnesota, Missouri, or Iowa, despite extensive collection efforts in those states. However, care should be taken to look for it and more work needs to be done to confirm the eastern extent of the range of *Agapostemonangelicus*. In areas where the two species may overlap, it is recommended that females identified morphologically as *Agapostemonangelicus* or *Agapostemontexanus* be treated as a single morphospecies. The two species can also be separated by DNA barcodes.^1^

#### ﻿ Agapostemon (Agapostemon) melliventris

Taxon classificationAnimaliaHymenopteraHalictidae

Cresson

2BE9D4EE-91AC-5E04-8770-7C82FF03F895

##### Diagnosis.

Female *Agapostemonmelliventris* can be recognized by having the apex of the clypeus yellow as well as their non-metallic, light-colored metasoma. The terga are generally amber-colored but can be dark enough (e.g., Fig. 4B) to resemble *Agapostemonvirescens*.

Male *Agapostemonmelliventris* can be recognized by having the metasoma primarily yellow (Fig. 9A), with just thin dark bands, and they also have the hind femur much skinnier (Fig. 8A) than any of the other species treated here.

##### Remarks.

*Agapostemonmelliventris* is not known from the midwestern US, though Roberts (1972) records if from eastern Nebraska and Kansas, so there is the potential for it to be found in Missouri and Iowa. We are not aware of any recent collections east of the 98^th^ Meridian.

#### ﻿ Agapostemon (Agapostemon) sericeus

Taxon classificationAnimaliaHymenopteraHalictidae

(Forster)

F638DA21-69D3-5C5F-B8A3-860D7F7E3F27

##### Diagnosis.

The female of *Agapostemonsericeus* can be recognized by the combination of the metallic green metasoma (as in Fig. 4C) and the reticulate sculpturing of the scutum (Figs 5B, 6A). It is most similar to *Agapostemonsplendens*, but *Agapostemonsplendens* has the scutum more punctured (Fig. 6B) rather than reticulate, and *Agapostemonsericeus* can be further distinguished by its sharply angled dorsolateral ridge of the pronotum (Fig. 5B) and by having the ventral pleural tubercle flush with the plate (Fig. 6C).

Male *Agapostemonsericeus* have S3 and S4 with a low transverse swelling and generally have distinct yellow marks on the apical sterna (Fig. 7C). They are most similar to males of *Agapostemontexanus* but can be distinguished by the relative lengths of F1 and F2: in *Agapostemonsericeus* F1 is slightly more than half the length of F2, whereas in *Agapostemontexanus* F1 is about three-fourths the length of F2 (Fig. 10). They can also be separated by the genitalia (see Roberts 1972).

##### Remarks.

*Agapostemonsericeus* was previously known as *Agapostemonradiatus* (Say) (e.g., Mitchell 1960; Roberts 1972) but was synonymized by Day and Fitton (1977).

Females of *Agapostemonfemoratus* Crawford, primarily a western species not recorded east of New Mexico, Colorado and Wyoming by Roberts (1972), are essentially identical to females of *Agapostemonsericeus*, though the males are quite distinct, possessing a grossly enlarged hind femur, its width and length equal or nearly so. The key in Roberts (1972) indicates the scutum of female *Agapostemonsericeus* is more distinctly punctate than *Agapostemonfemoratus*, but we do not consider this a reliable separating character. Curiously, there are several Missouri records of *Agapostemonfemoratus* from the 1960s identified by Roberts in separate online databases, (discoverlife.org, Ascher and Pickering 2022), but these were not included in his 1972 revision. We have not seen these specimens, but assume they represent mis-determined females of *Agapostemonsericeus*, not *Agapostemonfemoratus*.

#### ﻿ Agapostemon (Agapostemon) splendens

Taxon classificationAnimaliaHymenopteraHalictidae

(Lepeletier)

93483CC2-EAA6-59D6-BDCF-9FE3D3F52DF9

##### Diagnosis.

The female of *Agapostemonsplendens* can be recognized by the combination of the metallic green metasoma (as in Fig. 4C) and the coarsely punctured sculpturing of the scutum (Figs 5C, 6B). It is similar to *Agapostemonsericeus*, but that species has the sculpturing of the scutum more reticulate (Figs 4B, 6A). *Agapostemonsplendens* can be further distinguished by the obtuse dorsolateral ridge (Fig. 5C), the upraised ventral pleural tubercle (Fig. 6D), and it is generally larger than *Agapostemonsericeus* (though their sizes can intergrade).

Male *Agapostemonsplendens* can be recognized from all other midwestern *Agapostemon* by their very enlarged hind femur, with the length twice the width (Fig. 8C). They also tend to be larger than related species and have darker wings, but this character is subtle.

##### Remarks.

Some previous works (e.g., Mitchell (1960) and the keys on discoverlife.org) have used the shape of the ridges of the propodeal triangle (which often form a depressed medial triangle) to separate female *Agapostemonsplendens* from *Agapostemonsericeus* (which have parallel striae throughout the propodeal triangle), but we have found the character variable and it can be quite subtle, particularly in smaller *Agapostemonsplendens*. *Agapostemonsplendens* is largely restricted to areas of deep sands. We have examined material from throughout the range of *Agapostemonsplendens*, and there are many individuals, especially in the southern US, that have the scutal sculpturing more reticulate, similar to *Agapostemonsericeus*. More work is needed to determine whether this represents normal variation or is potentially due to a cryptic species complex.

#### ﻿ Agapostemon (Agapostemon) texanus

Taxon classificationAnimaliaHymenopteraHalictidae

Cresson

A6E0798F-9871-5171-A7DD-0B938367373F

##### Diagnosis.

The females of *Agapostemontexanus* have the metasoma metallic green (Fig. 4C) and can be recognized by the “double-punctured” scutum, which has a combination of intermixed large and small punctures (Fig. 5A). Females cannot be reliably distinguished from *Agapostemonangelicus*, so they should be separated based on range or DNA barcodes (see remarks for *Agapostemonangelicus* above).

Male *Agapostemontexanus* have S3 and S4 with a low transverse swelling and generally have distinct yellow marks on the apical sterna (Fig. 7B, D). They are extremely similar to *Agapostemonangelicus*, but *Agapostemontexanus* have the hind tibia with black stripes on the front and back (Fig. 8D), whereas *Agapostemonangelicus* has the hind tibia yellow anteriorly (Fig. 11). In addition, the two species can be separated based on the genitalia characters given in the key (Fig. 12), and at least in the midwestern US, the range of the two species largely does not overlap.

Male *Agapostemontexanus* are also similar to (and frequently misidentified as) *Agapostemonsericeus* but can be distinguished based on the relative lengths of F1 and F2: *Agapostemontexanus* has F1 about three-fourths the length of F2 (Fig. 10A), whereas *Agapostemonsericeus* has F1 slightly more than half the length of F2 (Fig. 10B).

##### Remarks.

*Agapostemontexanus* and *Agapostemonangelicus* largely do not overlap in range in the midwestern region, though Roberts (1972) reports *Agapostemonangelicus* from Iowa and eastern Kansas (see remarks under *Agapostemonangelicus*, above).

#### ﻿ Agapostemon (Agapostemon) virescens

Taxon classificationAnimaliaHymenopteraHalictidae

(Fabricius)

13AFF97E-73A0-502B-A92D-F6E0847010F0

##### Diagnosis.

Females of *Agapostemonvirescens* are the only midwestern species that has the metasoma dark (Fig. 4A), rather than metallic green (but see comments on dark *Agapostemonmelliventris*).

Males of *Agapostemonvirescens* can be recognized by the lack of a transverse swelling on S4 (Fig. 7A). In addition, S5 and S6 are usually all dark (Fig. 7A), whereas those sterna usually (though not always) have at least some yellow in other *Agapostemon* species. Finally, *Agapostemonvirescens* males have a relatively slender hind femur compared to most other midwestern *Agapostemon* species (see Fig. 8E).

##### Remarks.

Two western species with females with non-metallic metasomas have been recorded from nearby states though they have not been recorded from Iowa, Minnesota, or Missouri. *Agapostemonmelliventris* has been found as far east as eastern Kansas and Nebraska, but they have the metasoma lighter and the apex of the clypeus yellow, compared to black in *Agapostemonvirescens* females. In addition, *Agapostemoncoloradinus* (Vachal) is a Great Plains species which occurs as far east as eastern Kansas, though *Agapostemoncoloradinus* is usually noticeably larger than *Agapostemonvirescens* with finer, closer striations on the hypostomal area on the underside of the head (see Roberts 1972). Males of *Agapostemoncoloradinus* are similar to males of *Agapostemonvirescens*, but *Agapostemoncoloradinus* males have a dark stripe on the posterior surface of the hind femur and the inner gonostylar flap of *Agapostemoncoloradinus* lacks a pronounced, medially-directed process basally, which is present in *Agapostemonvirescens*.

#### ﻿Genus Augochlorella

Taxon classificationAnimaliaHymenopteraHalictidae

Sandhouse

E77DAE42-F4C7-5674-887B-3C57CDDFCA2A

##### Diagnosis.

The genus *Augochlorella* can be recognized by the combination of a normal oval-shaped tegula (as in Fig. 1B), the incomplete carina on the rear face of the propodeum (Fig. 2D), and the lack of a protruding paraocular lobe (Fig. 3D, F). Females lack the keel on S1 seen in *Augochlora* and they have simple hind tibial spurs. Males are quite similar to *Augochlora*, but *Augochlorella* males have the S4 apical margin weakly to strongly concave, versus straight in *Augochlora*. In addition, *Augochlorella* males lack distinct punctures on the rear of the propodeum (Fig. 2D) compared to *Augochlora* males which do have distinct punctures (Fig. 3B). Some *Augochlorella* are more of a greenish-bronze color.

### ﻿Keys to the midwestern species of *Augochlorella*

**Key to females**

**Table:** 

1	Propodeal triangle with striae continuing to posterior margin, or very nearly so (Fig. 13C–E); head round, only slightly broader than long (Fig. 13A); clypeus with apical fourth dark (Fig. 13A)	***aurata* (Smith)**
–	Propodeal triangle with striae not reaching posterior margin, leaving a distinct smooth portion (Fig. 13F–H); head slightly broader than long (Fig. 13B); clypeus with apical third dark (Fig. 13B)	***persimilis* (Viereck)**

**Key to males**

**Table:** 

1	Inner edge of hind basitarsus with hairs on apical two-thirds only slightly elongate, their length about equal to width of basitarsus (Fig. 13I); propodeal triangle with striae reaching posterior margin or nearly so (as in Fig. 13C–E)	***aurata* (Smith)**
–	Inner edge of hind basitarsus with hairs on apical two-thirds distinctly elongate, their length distinctly longer than width of basitarsus (Fig. 13J); propodeal triangle with striae not reaching posterior margin, leaving a smooth portion (as in Fig. 13F–H, though this character is subtle and less pronounced than in females)	***persimilis* (Viereck)**

**Figure 13.**
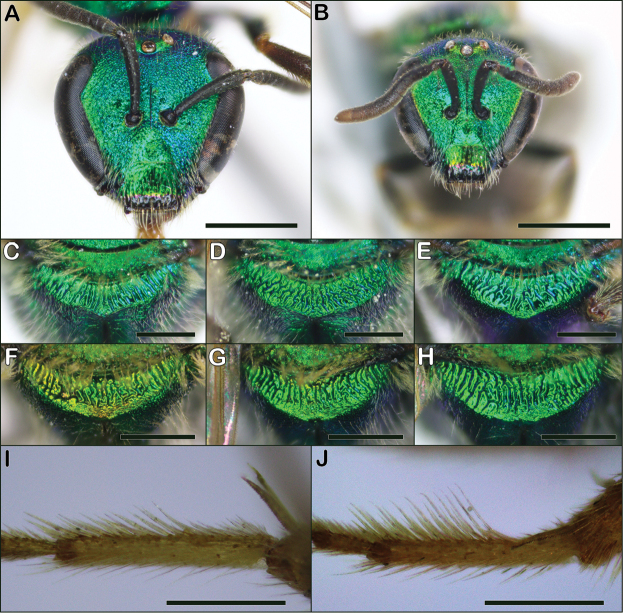
Augochlorella characters **A**Augochlorellaaurata female face **B**Augochlorellapersimilis female face **C–E**Augochlorellaaurata female propodeal triangles demonstrating the range of variation **C** apical margin carinate **D** apical margin largely lacking a carina **E** apical margin completely lacking a carina and striae partially removed from margin **F–H**Augochlorellapersimilis female propodeal triangles demonstrating the range of variation **F** propodeum with narrow smooth margin **G** propodeum with broad smooth margin **H** propodeum with narrow and irregular smooth margin **I**Augochlorellaaurata male hind basitarsus **J**Augochlorellapersimilis male hind basitarsus. Scale bars: 1 mm (**A, B**); 500 µm (**C–H, I, J**).

#### ﻿ Augochlorella aurata

Taxon classificationAnimaliaHymenopteraHalictidae

(Smith)

70239F90-EDEB-5503-B9E2-E93B9396FAD0

##### Diagnosis.

*Augochlorellaaurata* is very similar to *Augochlorellapersimilis*. Female *Augochlorellaaurata* can be recognized by having the striations of the propodeum continuing to the posterior margin (Fig. 13C–E), which often, but not always, is bordered by a carina (e.g., Fig. 13C). In contrast, females of *Augochlorellapersimilis* always have a distinct smooth portion before the margin of the propodeum (Fig. 13F–H). In addition, female *Augochlorellaaurata* are generally larger, have the head slightly longer and the apex of the clypeus is black only on the apical fourth (Fig. 13A). In contrast, *Augochlorellapersimilis* females are generally quite small, have the head slightly broader, and the apex of the clypeus is black on the apical third (Fig. 13B).

Female *Augochlorellaaurata* are also often confused with *Augochlorapura*, but *Augochlorellaaurata* have the paraocular lobes less protuberant (Fig. 3D, F) than *Augochlorapura*, and *Augochlorellaaurata* also lack a keel on S1.

Male *Augochlorellaaurata* can be separated from *Augochlorellapersimilis* by the hair on the apical two-thirds of the inner edge of the hind basitarsus, which is short in *Augochlorellaaurata*, with the length of the hairs about equal to the width of the basitarsus (Fig. 13I), whereas *Augochlorellapersimilis* has the hairs distinctly longer than the width of the basitarsus (Fig. 13J). In addition, the striae on the propodeal triangle of *Augochlorellaaurata* reach the posterior margin (as in Fig. 13C–E) whereas male *Augochlorellapersimilis* generally have a smooth portion before the margin.

Male *Augochlorellaaurata* are often confused with *Augochlorapura* males, but *Augochlorellaaurata* have the margin of S4 concave rather than straight, and they lack distinct punctures on the rear of the propodeum (Fig. 2D), compared to distinctly punctured in *Augochlorapura* (Fig. 3B).

##### Comments.

*Augochlorellaaurata* and *Augochlorellapersimilis* are often confused in collections and some females can intergrade to the degree where they are impossible to differentiate. Males are also frequently confused because the hind basitarsus character is often misinterpreted since both species have the basal third of the basitarsus with distinctly shorter hairs, which can cause confusion in keys that focus on the length of the basal hairs rather than the apical hairs, such as Coelho (2004), or the keys on discoverlife.org that incorrectly state that *Augochlorellaaurata* males have the “hair on rear basitarsus all about the same length”.

Given the high level of variation in *Augochlorellaaurata*, it seems likely that it is a species complex. Supporting this hypothesis are the various forms that Ordway (1966) recognized, one of which was elevated to species rank by Coelho (2004), as well as the high barcode diversity found in the species (Sheffield et al. 2009).

#### ﻿ Augochlorella persimilis

Taxon classificationAnimaliaHymenopteraHalictidae

(Viereck)

A9FE1A28-2F27-53EF-BB6F-B41BD5CF0890

##### Diagnosis.

*Augochlorellapersimilis* is very similar to *Augochlorellaaurata*. Females can be distinguished by the lack of rugae at the rear of the propodeal triangle, though this character can often be subtle (Fig. 13F–H). In addition, *Augochlorellapersimilis* tend to be smaller than *Augochlorellaaurata*, and they have a more extensive apical black mark on the clypeus, with the black part taking up approximately one-third of the length of the clypeus (Fig. 13B), compared to approximately one-fourth the length of the clypeus in *Augochlorellaaurata* (Fig. 13A). Note that there are often females of *Augochlorellapersimilis* and *Augochlorellaaurata* that cannot be reliably separated.

Male *Augochlorellapersimilis* can be separated from *Augochlorellaaurata* by the length of the hairs on the inner side of the hind basitarsus: *Augochlorellapersimilis* have the hairs very short for the basal third, then the hairs flare out to about twice the width of the basitarsus (Fig. 13J). In contrast, the hairs on *Augochlorellaaurata* are short for the basal third, and only get slightly longer, about equal in length to the width of the basitarsus (Fig. 13I). Like females, the males of *Augochlorellapersimilis* also have a lack of rugae at the rear of the propodeal triangle but it is less distinct (see Fig. 13F–H).

##### Comments.

This species has a more southern distribution than *Augochlorellaaurata*, though the species commonly overlap and co-occur. The northern extent of the range of *Augochlorellapersimilis* reaches the southern part of Michigan, Minnesota, and Wisconsin (Wolf and Ascher 2008; Gibbs et al. 2017).

#### ﻿Genus Augochlora

Taxon classificationAnimaliaHymenopteraHalictidae

Smith

4FE6DF87-0227-5476-86B5-61FC45E654E0

##### Comments.

*Augochlorapura* is the only species of *Augochlora* that occurs in the midwestern United States.

#### ﻿ Augochlora (Augochlora) pura

Taxon classificationAnimaliaHymenopteraHalictidae

(Say)

08C86848-7F67-5DD9-9487-910CFC28984D

##### Diagnosis.

*Augochlorapura* is most similar to *Augochlorellaaurata* and *Augochlorellapersimilis*. Both sexes of *Augochlorapura* can be recognized by the distinct and prominent facial lobes (Fig. 3C, E), which extend below the level of the base of the mandible and are stronger than those found in *Augochlorella*, but the difference is subtle and easy to confuse. Female *Augochlorapura* are unique in having a keel on S1 (Fig. 3A) and the hind tibial spur is simple. In addition, the mandibles of *Augochlorapura* females are more robust, with 2 distinct and nearly equally-sized apical teeth, whereas *Augochlorella* have 1 main tooth and a smaller subapical tooth.

Males of *Augochlorapura* can be further recognized from *Augochlorella* by their straight apical margin on S4 (compared to concave in *Augochlorella*) and they have distinct punctures on the rear of the propodeum (Fig. 3B), compared to impunctate or obscure punctures in *Augochlorella* males (Fig. 2D).

##### Comments.

*Augochlora* and *Augochlorella* are frequently confused in collections, especially males. Midwestern specimens of *Augochlorapura* fall under subspecies *Augochlorapurapura*. More work is needed to determine whether *Augochlorapurapura* and *Augochlorapuramosieri* Cockerell are distinct taxa.

#### ﻿Genus Augochloropsis

Taxon classificationAnimaliaHymenopteraHalictidae

Cockerell

F5899246-A152-5200-8B2B-812D14CF20E7

##### Diagnosis.

Both sexes of *Augochloropsis* are diagnosed by the unique shape of the tegula, which has the inner posterior margin hooked (Fig. 1A). Females have the inner hind tibial spur with multiple straight teeth compared to broad teeth in *Agapostemon* or untoothed spurs in *Augochlorella* and *Augochlora*. *Augochloropsis* males have a uniquely-shaped S4 (see Fig. 20C, H, M), with a median point and lateral arms, though the sternum is typically hidden. Though typically strongly metallic green, many individuals are metallic bluish or even purplish.

### ﻿Keys to the midwestern species of *Augochloropsis*

**Key to females**

**Table:** 

1	Vertex (in frontal view) rising above ocelli by at least one ocellar diameter (Fig. 14A); dorsolateral angle of pronotum strongly lamellate, lamella produced as a strong right angle or nearly so (Fig. 14D); terga dull, strongly tessellate, with surfaces appearing granular, even on apical rims of terga (Fig. 14G); sand obligate species	***humeralis* (Patton)**
–	Vertex (in frontal view) **not** rising above ocelli (Fig. 14B, C); dorsolateral angle of pronotum more weakly lamellate, lamella forming a very broad obtuse angle (Fig. 14E, F); tergal surfaces not strongly dull, at least somewhat shining (Fig. 14H, I); found in various habitats	**2**
2	T2 hair fringe on apical margin with thickened flattened hairs unlike the hairs elsewhere on T2, and arranged closely together and appearing like the teeth of a comb along the apical margin of T2 (Fig. 15A); T2 with small, close punctures (typically about 1–2 puncture widths apart), surface between punctures generally appearing weakly tessellate (Fig. 14H)	***metallica* (Fabricius)**
–	T2 hair fringe on apical margin with hairs identical to the hairs elsewhere on T2 (Fig. 15B); T1 and T2 punctures more widely separated, typically 2–4 puncture widths apart on T2, space between punctures strongly shining, without tessellation or other microsculpture (Fig. 14I)	***viridula* (Smith)**

**Key to males**

**Table:** 

1	Vertex (in frontal view) rising above ocelli by at least one ocellar diameter (Fig. 16A); dorsolateral angle of pronotum produced as a strong right angle or nearly so (Fig. 16D); terga dull, strongly tessellate, with surfaces appearing granular (Fig. 16G); sand obligate species	***humeralis* (Patton)**
–	Vertex (in frontal view) flattened, **not** rising above ocelli (Fig. 16B, C); dorsolateral angle of pronotum less pronounced and forming a very broad obtuse angle (Fig. 16E, F); tergal surfaces not strongly dull, at least somewhat shining (Fig. 16H, I); found in various habitats	**2**
2	T2 with apical fringe of distinctly thickened hairs (Fig. 16H); terga densely punctate, with punctures on T2 typically 1–2 puncture widths apart (Fig. 16H); propodeal triangle not as shiny, and more sculptured, than in *viridula*; genitalia with lateral margins of gonostyli parallel (Fig. 20F)	***metallica* (Fabricius)**
–	T2 with apical fringe composed of unthickened hairs (Fig. 16I); T2 surface shining (Fig. 16H), and T2 punctures more widely separated, typically 2–4 puncture widths apart; propodeal triangle shiny and less sculptured than in *metallica*; genitalia with lateral margins of gonostyli diverging apically (Fig. 20K)	***viridula* (Smith)**

**Figure 14.**
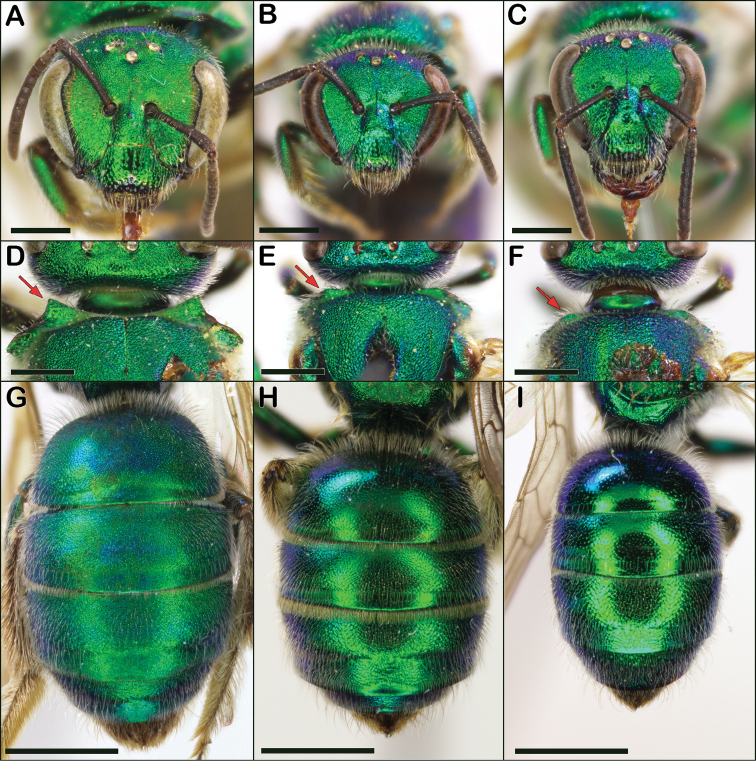
*Augochloropsis* female characters **A***Augochloropsishumeralis* face **B***Augochloropsismetallica* face **C***Augochloropsisviridula* face **D***Augochloropsishumeralis* well-developed, 90-degree pronotal flange indicated by red arrow **E***Augochloropsismetallica* obtuse pronotal flange indicated by red arrow **F***Augochloropsisviridula* obtuse pronotal flange indicated by red arrow **G***Augochloropsishumeralis* metasoma **H***Augochloropsismetallica* metasoma **I***Augochloropsisviridula* metasoma. Scale bars: 1 mm (**A–F**); 2 mm (**G–I**).

**Figure 15.**
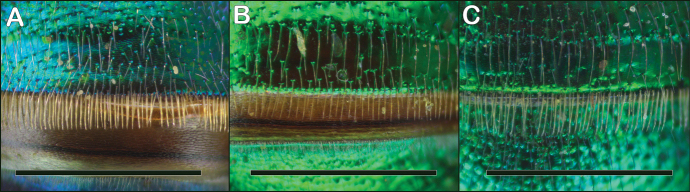
*Augochloropsis* female T2 hair fringes **A***Augochloropsismetallica* with thickened hairs **B***Augochloropsisviridula* with unthickened, slender hairs **C***Augochloropsis* sp. (likely undescribed species from Eastland, Texas) with intermediate hairs. Note the specimens in **A** and **B** have their metasomas stretched out, revealing the brown basal part of the tergum that is normally hidden under the preceding tergum; this was done to increase contrast of the hairs and make the differences clearer. Scale bars: 1 mm, all images at the same scale.

**Figure 16.**
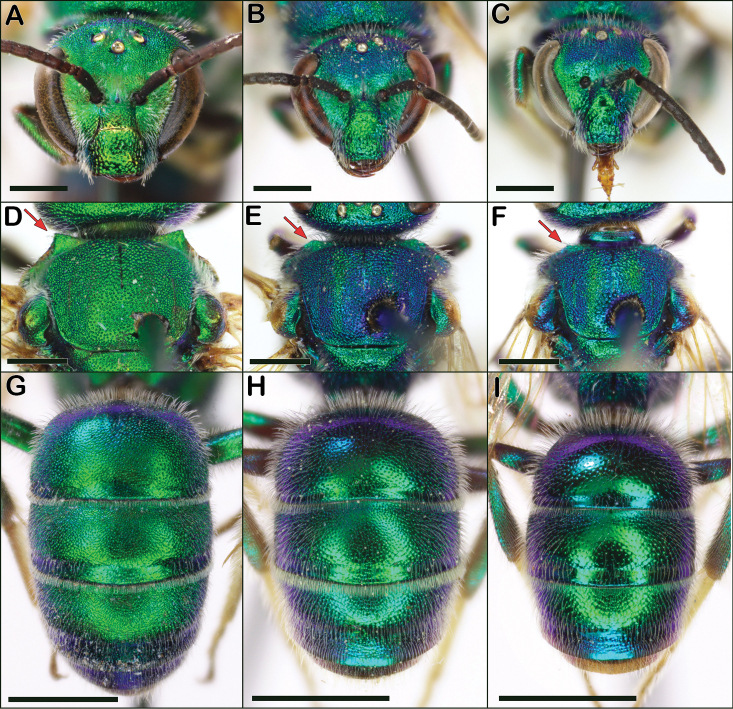
*Augochloropsis* male characters **A***Augochloropsishumeralis* face **B***Augochloropsismetallica* face **C***Augochloropsisviridula* face **D***Augochloropsishumeralis* well-developed, 90-degree pronotal flange indicated by red arrow **E***Augochloropsismetallica* obtuse pronotal flange indicated by red arrow **F***Augochloropsisviridula* obtuse pronotal flange indicated by red arrow **G***Augochloropsishumeralis* metasoma **H***Augochloropsismetallica* metasoma **I***Augochloropsisviridula* metasoma. Scale bars: 1 mm (**A–F**); 2 mm (**G–I**).

### ﻿Midwestern *Augochloropsis* species

#### ﻿ Augochloropsis (Paraugochloropsis) humeralis

Taxon classificationAnimaliaHymenopteraHalictidae

(Patton) stat. nov.

325809A4-9F43-55EC-9FC5-06CFB5C1657F

Augochlora humeralis Patton, 1879: 365 ♀♂. **Lectotype**: ♀ USA, North-western Kansas, 8 Sep 1877 leg. S.W. Williston, on goldenrod [ANSP]. Images examined by ZP and MA. **New lectotype designation.** (Labels read: “N.W. Kans. / Williston // Augochlora ♀ / humeralis n.s.”).
Agapostemon caeruleus Ashmead, 1890: 7 ♂ (not ♀) (syn. Sandhouse 1937). **Holotype**: ♂ USA, Colorado, Denver [USNM ENT 00536769]. Images examined by ZP and MA. Online record: http://n2t.net/ark:/65665/320b8ee01-69e8-40bd-ab90-fcb717151953. (Labels read: “Col. // [illegible symbol] Type / No 5516 / U.S.N.M. [red label] // Ashmead / Collection // Collection / Ashmead // Augochlora (Agapostemon) / ♀ coerulea Ash // USNM ENT / 00536769 [yellow label with barcode]”).
Augochlora sumptuosa bolliana Cockerell, 1909: 31 ♀ (syn. Under Augochloropsiscaerulea by Sandhouse 1937). Images cursorily examined by ZP and MA. **New synonym. Syntype(s?)**: USA, Texas, Lee Co. [USNM Type No. 23306 barcode #: 00536763]. Online record: http://n2t.net/ark:/65665/32fdc8c3b-b5b4-4cec-968e-4040825fa92d (Labels read: “Lee Co. / TX. 06 / VI. 0 [illegible symbol] // [red label] Type No. / 23306 / U.S.N.M. // A. sumptuosa / bolliana Ckll / TYPE // USNM ENT / 00536763 [yellow label with barcode]”).
Halictus (Augochlora) pattoni Vachal, 1903: 132 (proposed replacement name for humeralis Patton; syn. by Sandhouse 1937).
Augochlora (Augochloropsis) humeralis (in Titus 1901: taxonomy).
Augochloropsis caerulea (in Sandhouse 1937 [in part]: key; Lovell 1942: key).
Augochloropsis humeralis (in Dreisbach 1945: key).
Augochloropsis (Paraugochloropsis) sumptuosa (in Mitchell 1960 [in part]: key, redescription; Hurd 1979 [in part]: catalog; Moure and Hurd 1987 [in part]: catalog).

##### Diagnosis.

Both sexes of *Augochloropsishumeralis* can be distinguished from *Augochloropsismetallica* and *Augochloropsisviridula* by multiple characters. The pronotal flange of *Augochloropsishumeralis* has the lateral edges approaching 90 degrees (Figs 14D, 16D) whereas, the lateral edges of the pronotal flange in *Augochloropsismetallica* and *Augochloropsisviridula* are obtuse (Figs 14E, F, 16E, F, note they do still have a distinct pronotal flange as well). In addition, the vertex of *Augochloropsishumeralis* rises distinctly above the ocelli (Figs 14A, 16A) whereas it does not rise above the ocelli in *metallica* and *viridula* (Figs 14B, C, 16B, C). Finally, the strongly tessellate and “silky” texture of *Augochloropsishumeralis* (Figs 14G, 16G) is distinct in comparison to *Augochloropsismetallica* and *Augochloropsisviridula* (Figs 14H, I, 16H, I).

**Figure 17.**
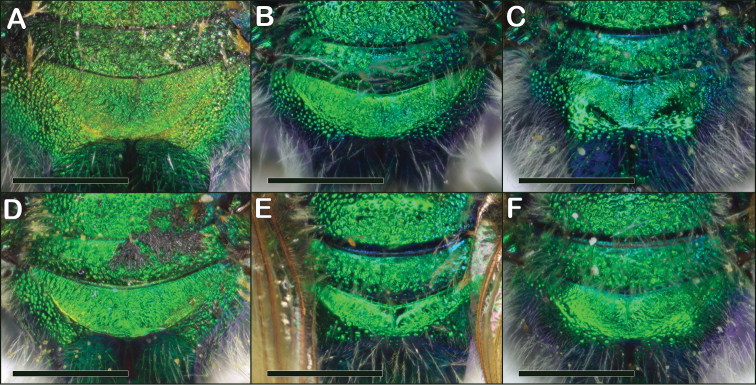
*Augochloropsis* female propodea **A***Augochloropsishumeralis***B***Augochloropsismetallica***C***Augochloropsisviridula***D***Augochloropsissumptuosa***E***Augochloropsisanonyma***F***Augochloropsis* sp. (likely undescribed species from Eastland, Texas). Scale bars: 1 mm, all images at the same scale.

*Augochloropsishumeralis* is similar in most respects to *Augochloropsissumptuosa*. The females can be separated by the more densely punctate metasomal terga: *Augochloropsishumeralis* has the punctures on T1 and T2 close together and separated by about one puncture width (at least over most of the terga), whereas *Augochloropsissumptuosa* has the punctures always well-separated (about 3–5 puncture widths apart). In addition, females of *Augochloropsissumptuosa* have a weak but distinct semicircular carina around the propodeal triangle (Fig. 17D), which *Augochloropsishumeralis* lacks (Fig. 17A). More work is needed on how to separate the males of *Augochloropsissumptuosa*, but the male of *Augochloropsishumeralis* appears to have the median emargination of S4 more acute (Fig. 20C), compared to more rounded truncate in *Augochloropsissumptuosa* (see Mitchell 1960: fig 111 (mislabeled as S5)), though this character is variable and it’s not clear how reliable it is.

##### Comments.

**Figure 18.**
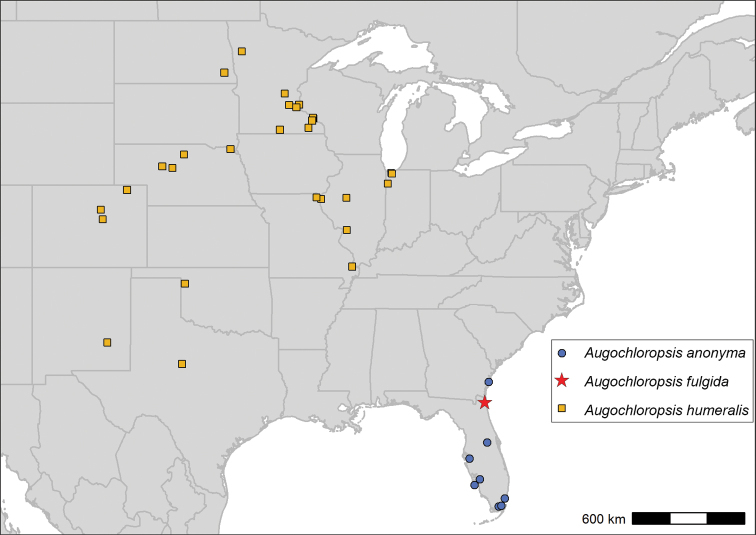
Map of specimens or observations examined for this study from the species *Augochloropsishumeralis*, *Augochloropsisanonyma*, and *Augochloropsisfulgida*.

What has previously been called *Augochloropsissumptuosa* by Mitchell (1960) is not a single species but rather a species complex. Therefore, we have reinstated the name *Augochloropsishumeralis* Patton for the species occurring in the midwestern United States and retained the name *Augochloropsissumptuosa* Smith for the species occurring in the southeastern United States. The exact extent of the range of *Augochloropsissumptuosa* is unclear, and it is not clear to what extent the ranges of *Augochloropsishumeralis* and *Augochloropsissumptuosa* may overlap. However, we have so far found no evidence that the two species overlap in range, with *Augochloropsissumptuosa* appearing to be limited to the east coast of the United States and *Augochloropsishumeralis* appearing to be limited to the prairie region (Fig. 18). Historic records of *Augochloropsissumptuosa* from Ohio were found to be misidentified *Augochloropsismetallica*. Since the identity of the midwestern species is clear, we have decided to proceed with a formal split; further delineation of the range of *Augochloropsissumptuosa* must be accomplished in future research.

Two syntypes of *Augochloropsishumeralis* (1 male and 1 female) were located in the ANSP collection, where Sandhouse (1937) reported examining them. Though the specimens are undated and not clearly labeled as type specimens, the labels indicate that they are from the type locality. In addition, the specimens bear labels stating “Augochlorahumeralis n.s.” and “Augochlorahumeralis n.sp.”. The combination of the little-used name, the “n. sp.”, the type locality, and the fact that Sandhouse (1937) considered these types, makes us confident that these are indeed Patton’s syntypes. As a result, we have designated the female as the lectotype, making the male a paralectotype. Additional paralectotypes may potentially be present at the Smithsonian, as Titus (1901) states “Mr. Ashmead very kindly examined specimens in the U.S.N.M. of *A.humeralis* Patt., marked ‘N. W. Kans., Williston’”. However, our inquiries to the Smithsonian have received no answer.

Sandhouse (1937) considered the name *Augochloropsiscaerulea* (Ashmead) to have priority because the name *humeralis* is a secondary homonym in the genus *Halictus*. However, *humeralis* is not a secondary homonym in the genus *Augochloropsis* and the substitute name is no longer in use, so following IZCN Article 59.3, the name *humeralis* is available and has priority.

*Augochlorasumptuosabolliana* Cockerell is from Texas and was synonymized with *Augochloropsissumptuosa* by Sandhouse (1937). Based on the online images of one of the syntypes, we are tentatively assigning it as a synonym of *Augochloropsishumeralis*, but a more critical evaluation of the specimen, with additional Texas material, should be performed.

##### Range.

*Augochloropsishumeralis* occurs throughout the prairie region, ranging from North Dakota and Minnesota south to New Mexico and Texas, extending to Colorado in the west and Indiana in the east (Fig. 18). Specimens from Indiana Dunes National Park represent the easternmost records.

##### Biology.

*Augochloropsishumeralis* is polylectic and nests are associated with deep sand (MA, pers. obs.). The sociality and the specifics of the nesting biology are unknown.

##### Material examined.

**Colorado: Adams Co.**: Denver (39.8207, -104.8613): 1 ♂ [iNat], 29 Aug 2019, @francesco167 leg.; **Douglas Co.**: (39.3467, -104.7511): 1 ♀ [iNat], Jul 2020, @calebcam leg.; **Logan Co.**: (40.7752, -103.2721): 1 ♂ [iNat], 22 Aug 2014, R. Webster leg. **Illinois: Hancock Co.**: Warsaw (40.3427, -91.4493): 1 ♂ [iNat], 14 Aug 2016, A. Moorehouse leg., *Monardapunctata*; **Madison Co.**: [MASR]; **Mason Co.**: (40.3921, -89.9104): 1 ♀ [iNat], 18 Jun 2019, A. Moorehouse leg., *Asclepias* sp. **Indiana: Lake Co.**: Indiana Dunes NP, Marquette Trail (41.6111, -87.2365): 1 ♀ [IDNP], 19 Jun 2019, McGill leg., blue pan; Indiana Dunes NP, Miller woods (41.6057, -87.2644): 1 ♂ [IDNP], 12 Sep 2018, McGill leg., white pan; 1 ♀ [IDNP], 4 Jun 2019, McGill leg., yellow pan; 1 ♀ [IDNP], 23 Jul 2019, McGill leg., yellow pan; Indiana Dunes NP, Miller woods (41.6071, -87.2644): 1 ♀ [IDNP], 23 Jul 2019, McGill leg., yellow pan; **Newton Co.**: Kankakee Sands (41.0848, -87.402): 1 ♀ [iNat], 24 May 2018, D. Lucas leg.; **Porter Co.**: Indiana Dunes National Lakeshore, Mnoke Prairie (41.6185, -87.1012): 1 ♀ [IDNP], 29 Jun 2017, J. Villalpando leg., bee bowl. **Minnesota: Faribault Co.**: (43.7, -93.96): 1 ♀ [UMSP], 18 Sep 1911; **Fillmore Co.**: Pin Oak SNA (43.79261, -92.21915): 1 ♀ [MNDNR], 24 Jul 2017, bowl; **Hennepin Co.**: (44.9, -93.4): 1 ♂ [UMSP], date unknown; **Norman Co.**: Agassiz Dunes SNA (47.51154, -96.28976): 1 ♂ [MNDNR], 24 Aug 2015, bowl; **Sherburne Co.**: Sherburne National Wildlife Refuge (45.46477, -93.67435): 2 ♀ [EERC], 15 Aug 2016, E. Evans leg., bowl; **Wabasha Co.**: Weaver Dunes (44.27746, -91.93892): 1 ♀ [UMSP], 28 May 2015, M.J. Hatfield leg., *Ceanothusherbaceus*; Weaver Dunes TNC/SNA (44.25096, -91.93795): 21 ♀ [MNDNR], 6 May 2017, bowl; 15 ♀ [MNDNR], 26 Jun 2017, bowl; 8 ♂ [MNDNR], 24 Jul 2017, bowl; 3 ♂ [MNDNR], 19 Aug 2017, bowl; 1 ♀ [MNDNR], 21 Sep 2017, bowl; **Washington Co.**: Belwin Conservancy (44.9241, -92.7931): 1 ♀ [EERC], 4 Sep 2015, J. Gardner leg., net, *Solidagonemoralis*; Belwin Conservancy (44.92569, -92.80001): 1 ♀ [CRC], 12 Jun 1995, C.C. Reed leg., net, *Penstemongrandifloris*; 1 ♀ [UMSP], 12 Jun 1995, C.C. Reed leg., net, *P.grandiflorus*; 9 ♀ [CRC], 15 Jun 1995, C.C. Reed leg., net, *P.grandifloris*; 6 ♀ [CRC], 16 Jun 1995, C.C. Reed leg., net, *P.grandifloris*; 1 ♀ 3 ♂ [CRC, UMSP], 15 Aug 1995, C.C. Reed leg., net, *Daleapurpurea*; 3 ♀ [CRC], 13 Jun 1997, C.C. Reed leg., net, *P.grandifloris*; Gray Cloud Dunes (44.79, -92.957): 1 ♀ 4 ♂ [UMSP], 9 Jul 1988; Grey Cloud Dunes (44.79, -92.957): 1 ♂ [CNBL], 23 Jul 2018, J. Petersen leg., net; Grey Cloud Dunes (44.7912, -92.9601): 1 ♂ [iNat], 14 Sep 2018, A. Birkey leg.; Grey Cloud Dunes SNA (44.79004, -92.95536): 1 ♂ [MNDNR], 9 Oct 2018, net, *S.nemoralis*; Grey Cloud Dunes SNA (44.790046, -92.955076): 1 ♂ [MNDNR], 31 Jul 2018, net, *D.villosa*; **Winona Co.**: Whitewater WMA (44.15033, -92.00066): 1 ♀ [MNDNR], 6 May 2017, bowl; 1 ♀ [MNDNR], 26 Jun 2017, bowl. **Missouri: Clark Co.**: [MASR]; **Scott Co.**: [MASR]. **Nebraska: Hooker Co.**: [MASR]; **Rock Co.**: (42.5, -99.8): 1 ♂ [iNat], Sep 2018, @allysond leg.; **Thomas Co.**: Neb Ntl For, near Halsey, 1 ♂ [WRME], 9 Aug 1991, Arduser leg.; Neb. Ntl For. Nr Whitetail campground: 1 ♀ [WRME], 10 Aug 1991, Arduser leg., *Helianthuspetiolarus*. **New Mexico: Chaves Co.**: [MASR]. **North Dakota: Ransom Co.**: (46.474534, -97.342645): 1 ♂ [iNat], 9 Aug 2021, E. Wood leg.. **Oklahoma: Ellis Co.**: [MASR]. **South Dakota: Clay Co.**: Missouri National Recreation River (42.76215, -96.9743): 1 ♂ [iNat], 7 Jul 2021, @stenthesnake leg. **Texas: Taylor Co.**: (32.32, -99.92): 1 ♀ [OSUC], 18 Jun 1952, J.N. Knull, D.J. Knull leg.

#### ﻿ Augochloropsis (Paraugochloropsis) metallica

Taxon classificationAnimaliaHymenopteraHalictidae

(Fabricius)

B0093FDA-70B2-5EFB-A0E8-DF014DEA3C33

**Figure 19.**
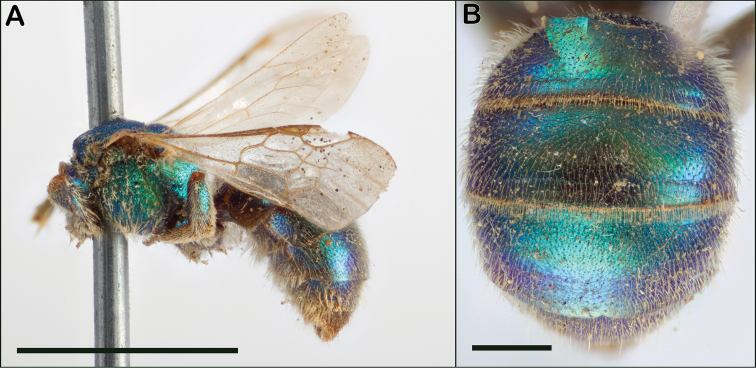
*Augochloropsismetallica* holotype female **A** lateral view **B** metasoma. Images provided by Lars Vilhelmsen, Sree Selvantharan, and Anders Illum of the Natural History Museum of Denmark, and used with permission. Scale bars: 5 mm (**A**); 1 mm (**B**).

Andrena metallica Fabricius, 1793: 309 ♀. **Holotype**: ♀ “America” [NHMD 308680]. Images examined by ZP and MA (Fig. 19) (Labels read: “metalli. / ??[line illegible] // NHMD / 308680 [label with QR code] // Megilla metallica F. / Syst. Piez. 1804: 332. 19 // TYPE [red label]”).
Augochlora fervida Smith, 1853: 81 ♂ (syn. [under cuprea] by Sandhouse 1937, syn. by Mitchell 1960 and Moure 1960). **Holotype**: ♂ North America [NHMUK014024969] Images examined by ZP and MA. Online record: https://data.nhm.ac.uk/object/3429259d-5af9-4c5f-9062-96a4a2770077 (Labels read: “Type / H.T. [label is circular with red border] // B.M. TYPE / HYM / 14.a.1230 // B.M. TYPE / HYM. / augochlora / fervida / smith 1853 // fervida / Type Sm. // Ent. Club. / 44-12. // NHMUK 014024969 [label with QR code]”).
Augochlora (Augochloropsis) cleomis Titus, 1901: 135 ♀♂ (syn. by Moure 1960). **Syntypes**: ♀♂ USA, Colorado, Horsetooth Gulch, near Ft. Collins. Not examined.
Halictus chorisis Vachal, 1903: 136 ♀ (syn. by Sandhouse 1937 [under cuprea], syn. by Mitchell 1960 [under metallica metallica]). **Lectotype**: ♀ USA, Georgia (designated by Moure and Hurd 1987). Not examined.
Megilla metallica (in Fabricius 1804: taxonomy).
Augochlora fervida (in Robertson 1895: taxonomy; Robertson 1902: key; Cockerell 1906: notes).
Augochloropsis cuprea (in Sandhouse 1937 [in part]: key; Lovell 1942: key; Dreisbach 1945: key).
Augochloropsis (Paraugochloropsis) metallica metallica (in Mitchell 1960: key, redescription; Hurd 1979: catalog; Moure and Hurd 1987: catalog).
Augochloropsis (Paraugochloropsis) metallica (in Stephenson et al. 2018: checklist; Camilo et al. 2018: checklist; Decker et al. 2020: checklist).

##### Diagnosis.

Both sexes of *Augochloropsismetallica* are most similar to *Augochloropsisviridula*, but *Augochloropsismetallica* can be separated from *Augochloropsisviridula* by the thicker hair fringe on the apical edge of T1 and T2; *Augochloropsismetallica* has the hairs noticeably thicker than the other hairs of the metasoma (Figs 14H, 15A, 16H), whereas the fringe hairs of *Augochloropsisviridula* are not noticeably thicker than the other metasomal hairs (Figs 14I, 15B, 16I). In addition, the terga of *Augochloropsismetallica* are more closely punctured, separated by about one puncture width on T2 (Fig. 14H, 16H), whereas the terga of *Augochloropsisviridula* are more sparsely punctured and separated by at least 2–4 puncture widths on T2 (Figs 14I, 16I).

Both sexes of *Augochloropsismetallica* can be separated from *Augochloropsishumeralis* by the shape of the pronotal flange and angle, which is smaller and has an obtuse lateral angle in *Augochloropsismetallica* (Figs 14E, 16E), compared to the larger flange and 90-degree lateral angle in *Augochloropsishumeralis* (Figs 14D, 16D). In addition, *Augochloropsismetallica* has the metasomal terga shining, with at most weak tessellation (Figs 14H, 16H), whereas *Augochloropsishumeralis* has the metasomal terga strongly and densely tessellate, resulting in dull, silky coloration (Figs 14G, 16G).

##### Comments.

The holotype of *Augochloropsismetallica* (Fig. 19) is missing its head, but the punctures and hair bands on the metasoma, combined with the locality of “America” (Moure (1960) states “probably middle eastern U.S.A.”) are sufficient to confirm its identity.

We define *Augochloropsismetallica* in a much more restricted sense than previous authors, who lumped multiple taxa under *Augochloropsismetallica* (e.g., Sandhouse 1937; Mitchell 1960). We are here splitting *Augochloropsismetallica* (as defined by Mitchell 1960) into five taxa: *A.metallica*, *A.cuprea*, *A.fulgida*, *A.fulvofimbriata*, and *A.viridula*. In the original revision of the *Augochloropsis* of the United States, Sandhouse (1937) lumped at least six distinct taxa under the name *Augochloropsiscuprea* (Table 1). One reason for this over-lumping appears to be that Sandhouse (1937) did not actually examine any of the type specimens, and synonymized many species based on the description alone or through correspondence. Examination of Sandhouse-determined material in the UMSP shows that she consistently lumped specimens of *Augochloropsismetallica*, *Augochloropsisviridula*, and a third unknown species together. Mitchell (1960) clearly recognized that *Augochloropsismetallica* and *Augochloropsisviridula* [as *Augochloropsismetallicafulgida*] were distinct, and it is unclear why he only split them into subspecies.

The traditional view that *Augochloropsismetallica* extends down through Mexico and Central America is almost certainly incorrect and merely an artifact of the erroneously broad definition of the species adopted by previous workers. Though we have examined relatively little material from south of the United States, the material we have examined has not matched any of the US *Augochloropsis* treated here. The synonymy of *Augochloropsisfulvofimbriata* Friese, described from Costa Rica, is almost certainly incorrect. The source of the synonymy of *Augochloropsisfulvofimbriata* was originally made by Sandhouse (1937), who synonymized the male of *Augochloropsisfulvofimbriata* under *Augochloropsisignita* and the female of *Augochloropsisfulvofimbriata* under *Augochloropsiscuprea*. However, Michener (1954), in his revision of the bees of Panama, treated *Augochloropsisfulvofimbriata* as valid, and though he did not mention the synonymy of *A.fulvofimbriata* under *Augochloropsiscuprea*, he did state about *A.fulvofimbriata*: “Sandhouse incorrectly placed this species in the synonymy if [sic] *ignita*.” Based on that, Michener (1954) clearly considered *Augochloropsisfulvofimbriata* a valid species (and he certainly would have been familiar with *Augochloropsismetallica*, which was then called *A.cuprea*). Moure (1960) did not list *Augochloropsisfulvofimbriata* as a synonym of *Augochloropsismetallicametallica*. However, following that, the works of Mitchell (1960), Hurd (1979), Moure and Hurd (1987), and Moure et al. (2007) all treat *Augochloropsisfulvofimbriata* as a synonym of *Augochloropsismetallicametallica*, but none of them indicate it is a new synonym, which suggests they were just carrying over the synonymy by Sandhouse (1937). Here, though we have not examined any material of *Augochloropsisfulvofimbriata*, we follow the classification of Michener (1954) who was the last worker to treat the species, and we formally treat *Augochloropsisfulvofimbriata* Friese (**stat. nov.**), as valid. This is supported by the findings of Celis and Cure (2017), who recognized *Augochloropsisfulvofimbriata* and even classified it in a separate subgenus than *Augochloropsismetallica* (*Augochloropsiss.s.* rather than *Paraugochloropsis*).

Another synonym with issues is *Augochloropsischorisis* Vachal, which was originally synonymized under *Augochloropsiscuprea* by Sandhouse (1937) and listed as a synonym of *Augochloropsismetallicametallica* by Michell (1960). The type series of *Augochloropsischorisis* contains specimens ranging from Georgia and Texas to Brazil (Rasmussen 2012), making it undoubtedly a composite series (Cockerell 1949). The specimen from Georgia was designated as a lectotype by Moure and Hurd (1987), who considered it a synonym of *Augochloropsismetallicametallica*. We have not been able to examine the lectotype which cannot be located at the National Museum of Natural History in Paris, France (A. Touret-Alby, pers. comm.) and we are nominally accepting the synonymy.

Augochlora (Augochloropsis) cleomis was described from a male and female specimen from near Fort Collins, Colorado (Titus 1901). The types were not examined and it is not entirely clear from the description whether it is a synonym of *Augochloropsismetallica* or *Augochloropsishumeralis*. It was originally synonymized with *Augochloropsiscuprea* by Sandhouse (1937) and was later synonymized with *Augochloropsismetallicametallica* by Moure (1960). Sandhouse (1937) did not examine any specimens and it is unclear whether Moure (1960) did. Regardless, it is likely a synonym of *Augochloropsismetallica*, so we are nominally accepting the synonym.

##### Biology.

*Augochloropsismetallica* is polylectic and nests in the ground. However, the specifics of the nesting biology and sociality are unknown. *Augochloropsismetallica* is often associated with sandy areas, and it has been found in natural habitats (e.g., native prairies, wetlands), as well as disturbed sites and urban areas.

##### Range.

*Augochloropsismetallica* occurs in the eastern states and across the Great Plains (Fig. 21A). Recent surveys (2009 to present) by MSA and co-workers in Oklahoma, Kansas and Nebraska have found *Augochloropsismetallica* throughout these states and further to the west (whereas *Augochloropsisviridula* is absent from those western areas).

##### Material Examined.

**Arkansas: Arkansas Co.**: [MASR]; **Faulkner Co.**: [MASR]; **Franklin Co.**: [MASR]; **Jackson Co.**: [MASR]; **Monroe Co.**: [MASR]; **White Co.**: [MASR]; **Woodruff Co.**: [MASR]. **Illinois: Calhoun Co.**: [MASR]; **Carroll Co.**: [MASR]; **Jasper Co.**: [MASR]; **Madison Co.**: [MASR]; **Marion Co.**: [MASR]; **Randolph Co.**: [MASR]; **Williamson Co.**: [MASR]. **Iowa: Jasper Co.**: [MASR]. **Kansas: Barton Co.**: [MASR]; **Bourbon Co.**: [MASR]; **Butler Co.**: [MASR]; **Chase Co.**: [MASR]; **Coffey Co.**: [MASR]; **Dickinson Co.**: [MASR]; **Douglas Co.**: (38.88, -95.29): 1 ♀ [UMSP], 11 Jun 1919, W.F. Hoffman leg.; 1 ♀ [UMSP], 2 Jul 1919, W.F. Hoffman leg.; **Geary Co.**: [MASR]; **Gove Co.**: [MASR]; **Greenwood Co.**: [MASR]; **Hodgeman Co.**: [MASR]; **Lane Co.**: [MASR]; **Lyon Co.**: [MASR]; **Morris Co.**: [MASR]; **Osage Co.**: [MASR]; **Pawnee Co.**: [MASR]; **Pottawatomie Co.**: [MASR]; **Reno Co.**: [MASR]; **Rice Co.**: [MASR]; **Riley Co.**: [MASR]; **Sheridan Co.**: [MASR]; **Thomas Co.**: [MASR]; **Trego Co.**: [MASR]. **Minnesota: Anoka Co.**: Bunker Hills Reg. Pk. (45.2176, -93.2898): 1 ♀ [EERC], 8 Jun 2015, J. Gardner leg., net, *Tradescantiaoccidentalis*; Bunker Hills Regional Park (45.2176, -93.2899): 1 ♀ [EERC], 13 Jul 2016, E. Evans leg., bowl; Bunker Pr. Dunes (45.21, -93.27): 1 ♀ [UMSP], 20 Jun 1947; Cedar Creek Ecosystem Science Reserve (45.4323, -93.1894): 2 ♀ [EERC], 22 May 2015, J. Gardner leg., bowl trap; Cedar Creek Nat. Hist. (45.402673, -93.202601): 1 ♂ [CRC], 1 Aug 1991, C.C. Reed leg., net, *Daleapurpurea*; 1 ♂ [UMSP], 20 Aug 1991, C.C. Reed leg.; Cedar Creek Natural History Area (45.402673, -93.202601): 1 ♀ [UMSP], 23 Jul 1986; 1 ♂ [UMSP], 30 Jul 1990; 1 ♀ [UMSP], 21 Sep 1992; 1 ♀ [UMSP], 15 Aug 1995; Cedar Creek Ecosystem Science Reserve (45.4037, -93.1834): 1 ♂ [EERC], 12 Aug 2015, J. Gardner leg., net, *D.villosa*; Helen Allison Savanna SNA (45.38454, -93.16319): 1 ♀ [MNDNR], 6 May 2017, bowl; Rum River Cent. Reg. Pk. (45.2907, -93.3811): 1 ♀ [EERC], 12 Jun 2015, E. Evans leg., net, *Amorphafruticosa*; **Anoka/Isanti Co.**: Cedar Creek Natural History Area (45.402673, -93.202601): 1 ♀ [UMSP], 15 Aug 2000; **Hennepin Co.**: Crow Hassan Park Reserve (45.2, -93.63): 2 ♀ [UMSP], 13 Jul 1995, C.C. Reed leg., net, *Astersericeus*; **Isanti Co.**: Cedar Creek Natural History Area (45.402673, -93.202601): 1 ♀ [UMSP], 29 Aug 1981; 1 ♀ [UMSP], 13 Jul 1991; 1 ♀ [UMSP], 14 Jul 1992; 1 ♀ [UMSP], 30 Sep 1992; 1 ♀ [UMSP], 11 Aug 1993; Irving & John Anderson County Park (45.4602, -93.0594): 1 ♂ [EERC], 20 Jul 2015, E. Evans leg., net, *Asclepiastuberosa*; **Lincoln Co.**: Hole in the Mountain (44.25680554, -96.29248338): 1 ♀ [UMSP], 15 Jun 2016, N. Pennarolla, J. Leone leg., bowl; 1 ♀ [UMSP], 27 Jun 2017, N. Pennarolla, J. Leone leg., bowl; Hole-in-the-Mountain TNC (44.2412, -96.29963): 2 ♀ [MNDNR], 6 Jun 2016, bowl; 1 ♀ [MNDNR], 27 Jun 2016, bowl; **Murray Co.**: (44.0709, -95.5718): 1 ♀ [CNBL], 29 Jun 2019, Bee Bowls; **Pipestone Co.**: Prairie Coteau SNA (44.1241, -96.15275): 1 ♀ [MNDNR], 6 Jun 2016, bowl; **Stearns Co.**: St. Cloud (45.44, -94.16): 1 ♀ [UMSP], 22 Jun 1967; **Yellow Medicine Co.**: Mound Spring Prairie SNA (44.74521, -96.42999): 6 ♀ [MNDNR], 6 Jun 2016, bowl. **Missouri: Barry Co.**: [MASR]; **Barton Co.**: [MASR]; **Benton Co.**: [MASR]; **Boone Co.**: Columbia (38.943, -92.333): 1 ♀ [OSUC], 19 Oct 1955, W.A. Dimmitt leg.; **Camden Co.**: [MASR]; **Douglas Co.**: [MASR]; **Franklin Co.**: [MASR]; **Harrison Co.**: [MASR]; **Howard Co.**: Fayette (39.141, -92.686): 9 ♂ [UMSP], 25 Sep 1966, D.B. Crockett leg.; **Jackson Co.**: [MASR]; **Jasper Co.**: [MASR]; **Jefferson Co.**: [MASR]; **Laclede Co.**: [MASR]; **Lafayette Co.**: [MASR]; **Linn Co.**: [MASR]; **Macon Co.**: [MASR]; **Mercer Co.**: [MASR]; **Miller Co.**: [MASR]; **Monroe Co.**: [MASR]; **Newton Co.**: [MASR]; **Pettis Co.**: [MASR]; **Ray Co.**: [MASR]; **Reynolds Co.**: [MASR]; **Saline Co.**: [MASR]; **Scott Co.**: [MASR]; **St. Clair Co.**: [MASR]; **St. Louis Co.**: [MASR]; **Ste. Genevieve Co.**: [MASR]; **Stoddard Co.**: [MASR]; **Sullivan Co.**: [MASR]; **Taney Co.**: [MASR]. **Nebraska: Co.**: Halsey (41.904, -100.27): 1 ♀ [UMSP], 3 Sep 1924, R.W. Dawson leg.; **Lancaster Co.**: [MASR]; **Richardson Co.**: [MASR]. **North Carolina: Wake Co.**: Raleigh (35.799, -78.617): 1 ♀ [UMSP], 26 May 1940; 1 ♂ [UMSP], 17 Nov 1940. **Ohio: Gallia Co.**: (38.82, -82.3): 1 ♀ [OSUC], 23 Aug 1942, C.H. Kennedy leg.; **Jackson Co.**: (39.01, -82.61): 1 ♂ [OSUC], 9 Aug 1942, J.E. Gillaspy leg.; 1 ♀ [OSUC], 9 Aug 1942, R.W. Strandtmann leg.; **Lawrence Co.**: (38.6, -82.52): 1 ♂ [OSUC], 8 Aug 1942, R.W. Strandtmann leg.; 4 ♀ 1 ♂ [OSUC], 9 Aug 1942, R.W. Strandtmann leg.; 6 ♀ 1 ♂ [OSUC], 23 Aug 1942, C.H. Kennedy leg.; **Muskingum Co.**: New Concord (39.995, -81.741): 1 ♀ [OSUC], 22 May 1975, C. Dasch leg. **Oklahoma: Ellis Co.**: [MASR]. **South Dakota: Co.**: Black Hills (43.96, -103.77): 1 ♀ [UMSP], 15–30 Jun 1931, F. Miller leg. **Texas: Dallas Co.**: (32.73, -96.8): 1 ♀ [UMSP], 14 May 1937, H.C. Knutson leg., *Marshalliacaespitosa*; **Smith Co.**: (32.39, -95.26): 1 ♀ [UMSP], May 1947, Barr leg. **Virginia: Arlington Co.**: (38.87, -77.09): 2 ♂ [UMSP], 20 Jul 1929, C.E. Michel leg.; **Fauquier Co.**: Warrentown (38.721, -77.799): 1 ♂ [UMSP], 28 Jul 1929, C.E. Michel leg.

#### ﻿ Augochloropsis (Paraugochloropsis) viridula

Taxon classificationAnimaliaHymenopteraHalictidae

(Smith) stat. nov.

79050CF9-D859-50A6-963B-8F9B9609075B

Augochlora viridula Smith, 1853: 81 ♂. **Holotype**: ♂ USA, New York, Trenton Falls [NHMUK 014024971]. Images examined by ZP and MA. Online record: https://data.nhm.ac.uk/object/10fb10b0-58d6-448c-b1b8-d3807ca35e0e (Labels read “Type / H.T. [label is circular with red border] // B.M. TYPE / HYM / 14.a.1232 // B.M. TYPE / HYM. / augochlora / viridula / smith 1853 // viridula / Type Sm // Ent. Club. / 44-12. // NHMUK 014024971 [label with QR code]”).
Augochlora lucidula Smith, 1853: 81 ♀ (syn. Patton 1879). **Holotype**: ♀ North America. Images examined by ZP and MA. Online record: https://data.nhm.ac.uk/object/9195d66b-dde0-4554-a11e-8352601fa232 (Labels read “Type / H.T. [label is circular with red border] // B.M. TYPE / HYM / 14.a.1233 // B.M. TYPE / HYM / augochlora / lucidula / Smith 1853 // lucidula / Type Sm. // Ent. Club. / 44-12 // NHMUK 014024972 [label with QR code]”).
Halictus (Augochlora) viridissimus Viereck, 1910: 688 (proposed replacement name for viridula Smith, preoccupied in Halictus).
Augochlora viridula (in Robertson 1895: taxonomy; Robertson 1902: key; Cockerell 1905: taxonomy).
Augochloropsis cuprea (in Sandhouse 1937 [in part]: key; Lovell 1942: key).
Augochloropsis viridula (in Dreisbach 1945: key).
Augochloropsis metallica (Fabricius) (in Eickwort 1969: generic revision, description of genitalia and other features).
Augochloropsis (Paraugochloropsis) metallica fulgida (in Mitchell 1960: key, redescription; Hurd 1979: catalog; Moure and Hurd 1987: catalog; Gibbs 2017: biology).
Augochloropsis (Paraugochloropsis) fulgida (in Stephenson et al. 2018: checklist; Camilo et al. 2018: checklist; Decker et al. 2020: checklist).

##### Diagnosis.

Both sexes of *Augochloropsisviridula* can be recognized primarily by the lack of a thickened hair fringe on T1 and T2; the hairs that are present along the margin are slender and the same size and width as the rest of the hairs on the terga (Fig. 15B). This is in contrast to the females of *Augochloropsismetallica* which have the fringe hairs noticeably thickened (Fig. 15A). In addition, the terga of *Augochloropsisviridula* are more shining and sparsely punctured, and this is most apparent on T1 and T2, with punctures on T2 separated by at least 2–4 puncture widths on T2 (Figs 14I, 16I). In contrast, *Augochloropsismetallica* has the punctures on T1 and T2 closer together (punctures typically separated by 1–2 puncture widths on T2) and the interspaces between the punctures have slight tessellation, though they are still somewhat shining (Figs 14H, 16H).

**Figure 20.**
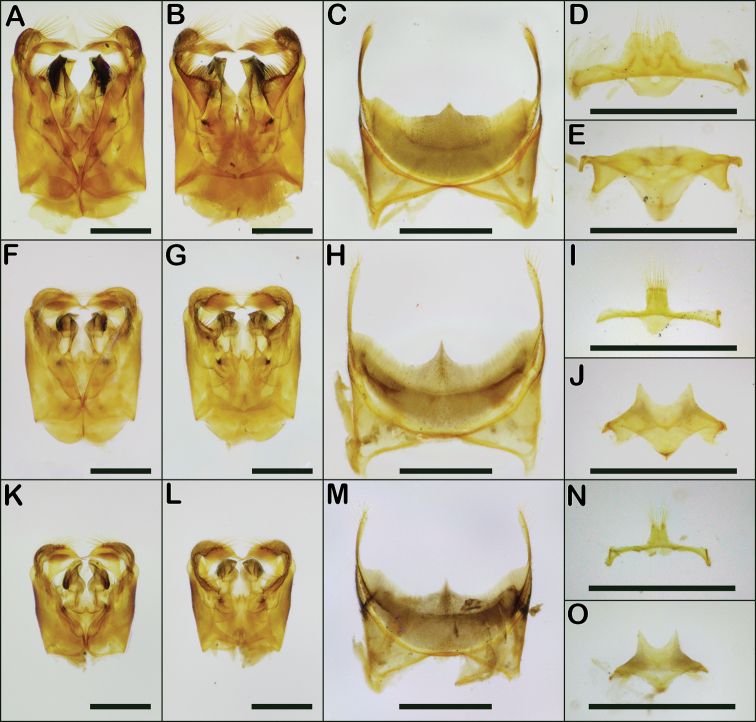
*Augochloropsis* male terminalia **A***Augochloropsishumeralis* dorsal genitalia **B***Augochloropsishumeralis* ventral genitalia **C***Augochloropsishumeralis* S4 **D***Augochloropsishumeralis* S8 **E***Augochloropsishumeralis* S7 **F***Augochloropsismetallica* dorsal genitalia **G***Augochloropsismetallica* ventral genitalia **H***Augochloropsismetallica* S4 **I***Augochloropsismetallica* S8 **J***Augochloropsismetallica* S7 **K***Augochloropsisviridula* dorsal genitalia (note the gonobase was torn off) **L***Augochloropsisviridula* ventral genitalia **M***Augochloropsisviridula* S4 **N***Augochloropsisviridula* S8 **O***Augochloropsisviridula* S7. Scale bars: 1 mm.

*Augochloropsisviridula* can be separated from *Augochloropsishumeralis* by its less developed pronotal flange, which is weak and forms an obtuse angle in *Augochloropsisviridula* (Figs 14F, 16F) compared to extensive and forming a right angle in *Augochloropsishumeralis* (Figs 14D, 16D), and *Augochloropsisviridula* has the terga smooth and shining (Figs 14I, 16I), compared to heavily tessellate and dull in *Augochloropsishumeralis* (Figs 14G, 16G).

*Augochloropsisviridula* can be separated from *Augochloropsisanonyma* and *Augochloropsisfulgida* by the relative lack of black hairs on the metasoma, having at most a few scattered black hairs on the apical terga (Fig. 14I), compared to copious black hairs over most of the terga in *Augochloropsisanonyma* and *Augochloropsisfulgida* (Fig. 23C, D).

##### Biology.

*Augochloropsisviridula* is a polylectic ground-nester. Nesting and sociality were documented by Gibbs (2017) [as *Augochloropsismetallicafulgida*]. Gibbs (2017) found a nest with two females, one of which had undeveloped ovaries and could have been a worker, suggesting that this bee may be primitively eusocial, however more work is needed to understand the degree of sociality of this species.

##### Range.

**Figure 21.**
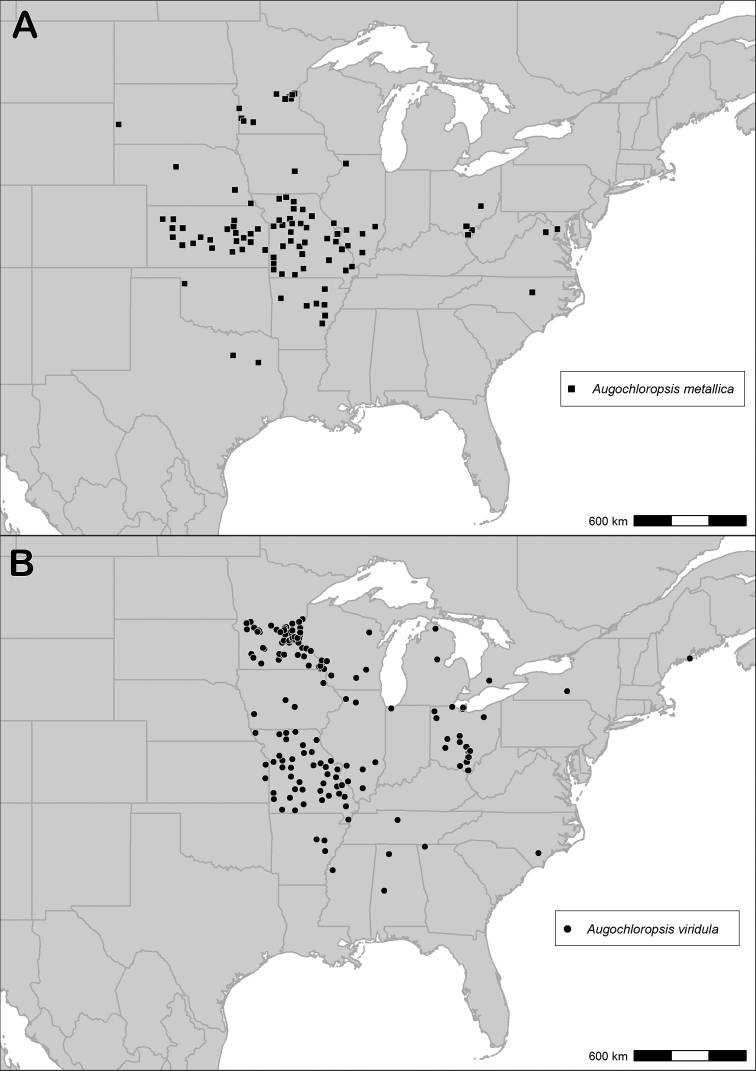
Map of specimens examined for this study **A***Augochloropsismetallica***B***Augochloropsisviridula*. Due to the limited geographic focus of our study, the easternmost extents of the ranges are relatively incomplete.

*Augochloropsisviridula* occurs throughout the eastern United States (Fig. 21B). It does not spread as far west as *Augochloropsismetallica* (see Fig. 21A), and recent surveys (2009 to present) by MSA and co-workers in Oklahoma, Kansas and Nebraska have found *viridula* only on the extreme eastern margins of these states, while *metallica* has been found throughout these states and further to the west. Mitchell records it [as *metallicafulgida*] extending south to Florida, though we have not evaluated material from the purported southernmost portion of the range.

##### Comments.

This species has historically been referred to as *Augochloropsismetallicafulgida* sensu Mitchell (1960). However, after examination of the holotype of *Augochloropsisfulgida* (Fig. 23), we found that it does not match the species concept used by Mitchell (1960) for *Augochloropsismetallicafulgida*. As a result, we resurrect the name *Augochloropsisviridula* (Smith), which was previously used by Robertson (1902) and Dreisbach (1945). *Augochloropsisfulgida* is reinstated as a separate species (see remarks for that species).

The species-level (rather than subspecies-level) recognition of *Augochloropsismetallica* and *Augochloropsisviridula* (previously classified as *Augochloropsismetallicametallica* and *Augochloropsismetallicafulgida*, respectively, by Mitchell (1960)) is supported by multiple characters, including the hair fringe on T2 (see Fig. 15A, B), difference in the degree of punctures and tessellation, the male terminalia, and differences in the extent of range. In particular, the male terminalia are distinct, with *Augochloropsismetallica* having the gonostyli more expanded (Fig. 20F, G), the gonocoxites more parallel-sided (Fig. 20F, G), the lateral arms of S4 more straight (Fig. 20H), and the lateral apodemes of S7 wider (Fig. 20J), in comparison, *Augochloropsisviridula* have the gonostyli narrower (Fig. 20K, L), the gonocoxites diverging apically (Fig. 20K, L), the lateral arms of S4 more curved (Fig. 20M), and the lateral apodemes of S7 narrower (Fig. 20O). The shape of S8 (Fig. 20I, N) appears to be too variable to be useful as a splitting character.

That *Augochloropsisviridula* (Smith) and *Augochloropsislucidula* (Smith) were different sexes of the same species was recognized by Patton (1879), Robertson (1895), and Cockerell (1905). However, both names were synonymized under *Augochloropsiscuprea* (along with *Augochloropsisanonyma*) by Sandhouse (1937). The name *Augochloropsisviridula* was then correctly applied by Dreisbach (1945). Mitchell (1960) clearly did not consider *viridula* and *lucidula* conspecific as he considered *Augochloropsisviridula* a junior synonym of *Augochloropsismetallicametallica* and *Augochloropsislucidula* a junior synonym of *Augochloropsismetallicafulgida*. Moure (1960) considered both *viridula* and *lucidula* junior synonyms of *Augochloropsismetallica*. Here, after examination of the primary types, we agree with the interpretation of Patton (1879) and Robertson (1895) in considering *Augochloropsisviridula* and *Augochloropsislucidula* as both conspecific and a true species.

In the generic revision of augochlorine bees by Eickwort (1969), the subspecies of *Augochloropsismetallica* were not recognized. However, the illustrations of the genitalia and other characters are clearly of *Augochloropsisviridula* (rather than *Augochloropsismetallica*) based on the apically diverging lateral margins of the gonostyli.

##### Material examined.

**USA: Alabama: Hale Co.**: [MASR]. **Arkansas: Lawrence Co.**: [MASR]; **Monroe Co.**: [MASR]; **White Co.**: [MASR]; **Woodruff Co.**: [MASR]. **Georgia: Catoosa Co.**: [MASR]. **Illinois: Carroll Co.**: [MASR]; **Jasper Co.**: [MASR]; **Madison Co.**: [MASR]; **Marion Co.**: [MASR]; **Ogle Co.**: (41.8751, -89.3474): 1 ♀ [NACH], 1 Jul 2017, B. Bruninga-Socolar leg., net, *Partheniumintegrifolium*; (41.896, -89.3461): 1 ♀ [NACH], 13 Jun 2017, B. Bruninga-Socolar leg., net, *Trifoliumpratense*; **Randolph Co.**: [MASR]; **Williamson Co.**: [MASR]. **Indiana: Lake Co.**: Indiana Dunes NP, Miller woods (41.6057, -87.2644): 1 ♀ [IDNP], 23 Aug 2019, McGill leg., blue pan. **Iowa: Clayton Co.**: [MASR]; **Jasper Co.**: [MASR]; **Pottawattamie Co.**: [MASR]; **Story Co.**: Ames (42.016, -93.624): 2 ♀ [UMSP], 16 Jun 1930, B.A. Haws leg., Swept from sweet clover. **Kansas: Johnson Co.**: [MASR]; **Linn Co.**: [MASR]. **Maine: Knox Co.**: (44.04, -69.04): 1 ♀ [OSUC], 15 Jul 1956, D.J. Borror leg. **Michigan: Cheboygan Co.**: (45.48, -84.49): 1 ♀ [OSUC], date unknown, C.H. Kennedy leg.; **Gladwin Co.**: [MASR]. **Minnesota: Anoka Co.**: Bunker Hills Regional Park (45.2143, -93.2797): 1 ♀ [EERC], 24 Jun 2016, J. Gardner leg., net, *Crepistectorum*; Cedar Creek Nat. Hist. (45.402673, -93.202601): 1 ♂ [UMSP], 1 Aug 1991, C.C. Reed leg.; Cedar Creek Natural History Area (45.402673, -93.202601): 1 ♀ [UMSP], 10 May 1993; Rum River Cent. Reg. Pk. (45.28686656, -93.37669731): 1 ♀ [EERC], 12 Jun 2015, E. Evans leg., net, *Rosaarkansana*; Rum River Cent. Reg. Pk. (45.2883, -93.38): 2 ♀ [EERC], 12 Jun 2015, E. Evans leg., net, *Ziziaaurea*; Rum River Cent. Reg. Pk. (45.2907, -93.3811): 7 ♀ [EERC], 12 Jun 2015, E. Evans leg., net, *Amorphafruticosa*; **Anoka/Isanti Co.**: Cedar Creek Natural History Area (45.402673, -93.202601): 1 ♀ [UMSP], 17 Sep 2004; **Blue Earth Co.**: Gilfillan Lake WMA (44.21091, -93.8494): 1 ♀ [MNDNR], 3 Oct 2016, net, *Symphyotrichumlanceolatum*; Maple River WMA (43.979867, -94.042629): 1 ♂ [MNDNR], 14 Aug 2015, net, *Solidagoaltissima*; **Carver Co.**: Schneewind WMA (44.80941, -93.82892): 1 ♀ [MNDNR], 8 Aug 2018, net, *Melilotusalba*; Schneewind WMA (44.80952, -93.82793): 1 ♀ [MNDNR], 16 Jul 2018, bowl; **Chisago Co.**: Wild River SP (45.5215, -92.7309): 1 ♀ [MNDNR], 22 Jun 2020, N. Gerjets leg., pantrap; **Douglas Co.**: StaffansonTNC (45.81606, -95.74604): 1 ♀ [CNBL], 5 Jun 2018, G. Pardee leg., net, *Z.aptera*; 1 ♀ [CNBL], 5 Jun 2018, I. Lane leg., net, *Z.aptera*; **Fillmore Co.**: (43.7, -92.2): 1 ♀ [UMSP], 24 May 1937, G. Kohls leg.; **Goodhue Co.**: Frontenac (44.53, -92.351): 1 ♀ [UMSP], 29 May 1930, C.E. Michel leg.; Spring Creek Prairie SNA (44.55522, -92.59502): 1 ♂ [MNDNR], 11 Aug 2017, net, *Asclepiasverticillata*; **Goodhue/Wabasha Co.**: E Frontenac, Lake Pepin (44.53, -92.351): 1 ♀ [UMSP], 29 May 1941, M.W. Wing leg.; Frontenac, Lake Pepin (44.53, -92.351): 1 ♀ [UMSP], 29 May 1941, M.W. Wing leg., net; **Hennepin Co.**: (44.9, -93.4): 1 ♀ [UMSP], 27 May 1922, A.A. Nichol leg.; Crow-Hassan Park Reserve (45.2018, -93.6311): 1 ♀ [MNDNR], 25 Aug 2015, bowl; Minnesota Valley National Wildlife Refuge (44.79892, -93.38589): 1 ♀ [MNDNR], 17 Jul 2017, net, *Solanumdulcamara*; St Bonifacius: 6 Mile Marsh (44.9113, -93.71958): 1 ♀ [CNBL], 28 Jul 2018, Z. Portman leg., net, *M.alba*; St Bonifacius: 6 Mile Marsh (44.9121, -93.7217): 1 ♀ [CNBL], 5 Jun 2020, Z. Portman leg., net, *Z.aurea*; **Houston Co.**: (43.67, -91.5): 3 ♀ [UMSP], 21 May 1938, H.E. Milliron leg.; (43.68, -91.47): 2 ♀ [UMSP], 23 May 1936, C.E. Michel leg.; 4 ♀ [UMSP], 23 May 1936, D. Murray leg.; 1 ♀ [UMSP], 23 May 1936, O. Elster leg.; 3 ♀ [UMSP], 23 May 1936, R. Cottrell leg.; 1 ♀ [UMSP], 22 May 1937, H.S. Telford leg.; 2 ♀ [UMSP], 24 May 1937, C.E. Michel leg.; 1 ♀ [UMSP], 20 May 1938, P. Nicholson leg.; 3 ♀ [UMSP], 21 May 1938, C.E. Michel leg.; 2 ♀ [UMSP], 21 May 1938, H.E. Milliron leg.; 1 ♀ [UMSP], 21 May 1938, R. Anderson leg.; 1 ♀ [UMSP], 22 May 1938, R. Anderson leg.; 1 ♀ [UMSP], 21 Jun 1938, C.E. Michel leg.; 1 ♀ [UMSP], 26 May 1940, I. Tarshie leg.; Beaver Crk. Valley St. Park (43.642, -91.581): 2 ♀ [UMSP], 4 Jul 1973, Malaise trap; Eitzen (43.51, -91.46): 1 ♀ [UMSP], 23 May 1936; Mississippi Bluff, 1–2 m N State Line (43.524, -91.28): 1 ♀ [UMSP], 30 May 1941, J.H. Hughes leg.; 1 ♀ [UMSP], 27 May 1950; 1 ♀ [UMSP], May 1957; Mississippi Bluffs 1 mi N. New Albin, Ia. (43.514, -91.279): 1 ♀ [UMSP], 29 May 1960; Mound Prairie SNA (43.76248, -91.42277): 1 ♀ [MNDNR], 26 Jun 2017, bowl; S.E. tip of county (43.52, -91.29): 1 ♀ [UMSP], 24 May 1935, H. Dodge leg.; Winnebago Cr. Vy., 2–4 m NE Eitzen (43.541, -91.415): 1 ♀ [UMSP], 27 Jun 1956; **Isanti Co.**: Cedar Creek Natural History Area (45.402673, -93.202601): 1 ♀ [UMSP], 1 Aug 1985; 2 ♀ [UMSP], 21 May 1987, *Rubus* sp; 1 ♂ [UMSP], 30 Sep 1992; 1 ♀ [UMSP], 1 Sep 1993; 2 ♀ [UMSP], 27 Jul 1994; 1 ♂ [UMSP], 17 Sep 1994; 1 ♀ [UMSP], 19 Aug 2000; 1 ♀ [UMSP], 19 Aug 2001; **Jackson Co.**: Des Moines River SNA (43.79222, -95.09111): 6 ♀ [MNDNR], 6 Jun 2016, bowl; **Kanabec Co.**: Rice Creek WMA (45.7389, -93.2044): 1 ♀ [MNDNR], 30 Jun 2020, D. Drons leg., net, *Rhusglabra*; **Kandiyohi Co.**: Brenner (45.4006, -95.2462): 1 ♀ [CNBL], 7 Jun 2018, G. Pardee leg., net, *Z.aptera*; Brenner Lake WPA (45.39926, -95.24568): 2 ♀ [MNDNR], 6 Jul 2016, bowl; Nelson (45.35289989, -95.11923718): 1 ♀ [CNBL], 26 Jun 2017, R. Tucker leg., net, *Cirsiumarvense*; 1 ♀ [CNBL], 4 Jun 2018, I. Lane leg., net, *Z.aurea*; 3 ♀ [CNBL], 4 Jun 2018, S. Marconie leg., net, *Z.aurea*; 1 ♀ [CNBL], 4 Jun 2018, T. Eicholz leg., net, *Z.aurea*; Rudningen (45.32725, -95.17902): 1 ♀ [CNBL], 26 Jun 2017, C. Herron-Sweet leg., net, *Achilleamillefolium*; **Le Sueur Co.**: Dove Lake WMA (44.22547, -93.7065): 1 ♀ [MNDNR], 1 Sep 2017, net, *So. Sp*; Kasota Prairie SNA (44.26502, -94.00384): 1 ♀ [MNDNR], 6 May 2017, bowl; **Lyon Co.**: Glynn Prairie SNA (44.2637757, -95.69623097): 1 ♀ [UMSP], 20 Jul 2017, N. Pennarolla, J. Leone leg., bowl; **Mille Lacs Co.**: Kunkel WMA (45.5741, -93.6623): 1 ♀ [MNDNR], 24 Jun 2020, D. Drons leg., pantrap; Princeton (45.571, -93.578): 1 ♀ [UMSP], 3 Oct 1994, A. Johnson leg.; **Murray Co.**: (44.0709, -95.5718): 1 ♀ [CNBL], 29 Jun 2019, Bee Bowls; **Olmsted Co.**: Oronoco Prairie SNA (44.14002349, -92.48913144): 1 ♀ [MNDNR], 13 Sep 2013, bowl; **Pine Co.**: Chengwatana State Forest (45.819, -92.7864): 1 ♂ [MNDNR], 16 Jul 2020, N. Gerjets leg., net, *Veranicastrumvirginicum*; St. Croix SP (45.9543, -92.5799): 1 ♀ [MNDNR], 25 Aug 2020, N. Gerjets leg., net, *So.* Sp.; **Pope Co.**: Glacial Lakes State Park (45.541, -95.531): 1 ♂ [UMSP], 25 Jul 1973, Malaise trap; **Ramsey Co.**: Bald Eagle Otter Lk. Reg. Pk. (45.09588474, -93.0494575): 1 ♀ [EERC], 5 Sep 2015, E. Evans leg., bowl trap; Battle Creek Reg. Pk. (44.9346, -93.0141): 1 ♀ [EERC], 10 Jun 2015, J. Gardner leg., net, *Cornussericea*; Battle Creek Reg. Pk. (44.93505, -93.015467): 2 ♀ [EERC], 27 May 2015, J. Gardner leg., net, *Geraniummaculatum*; Battle Creek Regional Park (44.9345, -93.013): 1 ♀ [EERC], 17 May 2016, E. Evans leg., bowl trap; Battle Creek Regional Park (44.94, -93.001): 2 ♀ [EERC], 8 Jun 2016, J. Gardner leg., net, *Ru. Allegheniensis*; Roseville, 3035 Fairview Avenue N (45.03262, -93.17757): 1 ♀ [UMSP], 5–7 Sep 2014, R.W. Holzenthal leg.; St Anthony Park (44.98, -93.2): 1 ♀ [UMSP], Jun year unknown; **Redwood Co.**: Cedar Mountain SNA (44.50489, -94.89886): 12 ♀ [MNDNR], 6 Jul 2016, bowl; **Renville Co.**: Morton Outcrops SNA (44.5501, -94.9902): 1 ♀ [MNDNR], 6 Jul 2016, bowl; 1 ♀ [MNDNR], 18 Jul 2016, bowl; **Sherburne Co.**: Sherburne National Wildlife Refuge (45.4973, -93.6851): 1 ♀ [EERC], 10 Jun 2016, E. Evans leg., net, *R.arkansana*; Uncas Dunes SNA (45.42750645, -93.69554017): 1 ♀ [MNDNR], 11 Jun 2013, net; **Stearns Co.**: Avon Hills Forest SNA (45.63589, -94.50259): 1 ♂ [MNDNR], 13 Sep 2018, net, *So.altissima*; St. Cloud (45.44, -94.16): 2 ♀ [UMSP], 25 May 1968; 1 ♂ [UMSP], 30 Jul 1968; **Stevens Co.**: Freeman WMA (45.46042, -95.97334): 1 ♀ [MNDNR], 21 Jun 2015, net, *R.* sp; Verlyn Marth Memorial Prairie SNA (45.7451, -96.00017): 3 ♀ [MNDNR], 6 Jul 2016, bowl; **Swift Co.**: Rice WPA (45.34486541, -95.32010344): 1 ♀ [UMSP], 26 Jun 2016, N. Pennarolla, J. Leone leg., bowl; **Wabasha Co.**: Reads Landing (44.402, -92.08): 1 ♀ [UMSP], 22 Jun 1934, C.E. Michel leg.; **Washington Co.**: (45.04, -92.89): 1 ♀ [UMSP], 9 May 1959; Afton State Park (44.846, -92.789): 1 ♀ [CRC], 11 Sep 1992, C.C. Reed leg., net; Arcola Bluffs SAC (45.1209, -92.7509): 1 ♀ [CNBL], 31 May 2018, K. Friedrich leg., vac, *G.maculatum*; 1 ♀ [CNBL], 14 Jun 2018, K. Friedrich leg., vac, *Erigeronphiladelphicus*; Big Marine Park Res. (44.2014, -92.8796): 6 ♀ [EERC], 7 Jun 2016, J. Gardner leg., net, *R.woodsii*; Lost Valley Prairie SNA (44.80086892, -92.81775955): 1 ♀ [MNDNR], 13 Sep 2013, bowl; Lost Valley SNA (44.802885, -92.823067): 1 ♀ [UMSP], 19 Sep 1990, C.C. Reed leg.; 1 ♀ [CRC], 19 Sep 1990, C.C. Reed leg., net; 1 ♀ [UMSP], 28 Jul 1992, C.C. Reed leg.; St. Croix Savanna SNA (45.00322082, -92.78344361): 1 ♀ [MNDNR], 16 Sep 2013, bowl; St. Croix Savanna SNA (45.00540834, -92.78347343): 1 ♀ [MNDNR], 13 Sep 2013, bowl; St. Croix Savanna SNA (45.006475, -92.785823): 1 ♀ [UMSP], 5 Aug 1994, C.C. Reed leg., *Monardafistulosa*; **Winona Co.**: Great River Bluffs SP (43.93895, -91.4113): 1 ♀ [MNDNR], 19 Aug 2017, bowl; **Wright Co.**: Lake Maria SP (45.31787, -93.93487): 1 ♀ [MNDNR], 6 May 2017, bowl. **Mississippi: Bolivar Co.**: Cleveland (33.741, -90.742): 1 ♀ [UMSP], 21 Apr 1937, R.W. Dawson leg. **Missouri: Atchison Co.**: [MASR]; **Barry Co.**: [MASR]; **Barton Co.**: [MASR]; **Benton Co.**: [MASR]; **Bollinger Co.**: [MASR]; **Callaway Co.**: [MASR]; **Camden Co.**: [MASR]; **Crawford Co.**: [MASR]; **Dallas Co.**: [MASR]; **Dent Co.**: [MASR]; **Douglas Co.**: [MASR]; **Franklin Co.**: [MASR]; **Greene Co.**: [MASR]; **Grundy Co.**: [MASR]; **Harrison Co.**: [MASR]; **Jackson Co.**: [MASR]; **Jasper Co.**: [MASR]; **Jefferson Co.**: [MASR]; **Johnson Co.**: [MASR]; **Laclede Co.**: [MASR]; **Lafayette Co.**: [MASR]; **Lewis Co.**: [MASR]; **Lincoln Co.**: [MASR]; **Macon Co.**: [MASR]; **Madison Co.**: [MASR]; **Mercer Co.**: [MASR]; **Monroe Co.**: [MASR]; **Montgomery Co.**: [MASR]; **Pemiscot Co.**: [MASR]; **Pettis Co.**: [MASR]; **Putnam Co.**: [MASR]; **Randolph Co.**: [MASR]; **Ray Co.**: [MASR]; **Reynolds Co.**: [MASR]; **Saline Co.**: [MASR]; **Shannon Co.**: [MASR]; **St. Francis Co.**: [MASR]; **St. Louis Co.**: [MASR]; **Ste. Genevieve Co.**: [MASR]; **Stoddard Co.**: [MASR]; **Taney Co.**: [MASR]; **Warren Co.**: [MASR]. **New York: Tompkins Co.**: Ithaca (42.442, -76.501): 1 ♀ [OSUC], 27 Aug 1950, J. Cillie leg. **North Carolina: Sampson Co.**: Ivanhoe (34.58, -78.25): 1 ♀ [UMSP], 3 May 1945, T.B. Mitchell leg. **Ohio: Champaign Co.**: (40.13, -83.77): 1 ♂ [OSUC], 24 Jul 1954; 1 ♀ [OSUC], 8 Jun 1994, N.F. Johnson leg., Malaise trap; **Delaware Co.**: (40.27, -83.01): 1 ♀ [OSUC], 2 Aug 1942, R.W. Strandtmann leg.; **Fairfield Co.**: (39.75, -82.63): 1 ♀ [OSUC], 16 Jun 1994, A. Sharkov leg.; **Franklin Co.**: (39.97, -83.01): 1 ♂ [OSUC], 21 Aug 1942; 1 ♀ [OSUC], 18 Jun 1952; **Greene Co.**: [MASR]; (39.69, -83.89): 1 ♀ [OSUC], 6 Jun 1956, J.N. Knull leg.; 1 ♀ [OSUC], 20 Jun 1957, J.N. Knull, D.J. Knull leg.; **Hocking Co.**: (39.49, -82.48): 1 ♀ [OSUC], 10 May 1935, R.C. Osburn leg.; 1 ♀ [OSUC], 14 Jun 1943, R.C. Osburn leg.; 1 ♀ [OSUC], 23 May year unknown, J.N. Knull, D.J. Knull leg.; 1 ♀ [OSUC], 14 Jun year unknown, R.C. Osburn leg.; **Jackson Co.**: (39.01, -82.61): 1 ♀ [OSUC], 9 Aug 1942, R.W. Strandtmann leg.; **Lawrence Co.**: (38.6, -82.52): 1 ♂ [OSUC], 8 Aug 1942, R.W. Strandtmann leg.; **Logan Co.**: (39.54, -82.41): 1 ♀ [UMSP], 16 Jul 1930, J. Patton leg.; **Lucas Co.**: [MASR]; (41.68, -83.47): 1 ♀ [OSUC], 19 May 2003, M. Arduser leg., *Lupinusperennis*; **Ottawa Co.**: Catawba Island (41.579, -82.836): 1 ♀ [OSUC], 27 Jun 1902, J.G. S. leg.; Put-in-Bay (41.649, -82.816): 1 ♀ [OSUC], 20–30 Jun 1924; 1 ♀ [OSUC], 14 Jul 1935, R.C. Osburn leg.; 1 ♀ [OSUC], 22 Aug 1941, R.C. Osburn leg.; 1 ♀ [OSUC], date unknown, C.H. Kennedy leg.; **Paulding Co.**: Charloe (41.131, -84.434): 1 ♀ [OSUC], 12 May 1951, H.F. Price leg.; **Scioto Co.**: (38.82, -82.99): 1 ♀ [OSUC], 6 Aug 1942, R.W. Strandtmann leg.; 1 ♀ [OSUC], 9 Jun 1943, J.N. Knull, D.J. Knull leg.; **Summit Co.**: Ira (41.182, -81.585): 1 ♀ [OSUC], date unknown, J.S. Hine leg.; **Vinton Co.**: (39.25, -82.49): 1 ♀ [OSUC], 20 Jun 1901; **Williams Co.**: Bryan (41.472, -84.553): 2 ♀ [OSUC], date unknown. **Tennessee: Davidson Co.**: Nashville: [MASR]. **Wisconsin: Burnett Co.**: (43.5, -88.71): 1 ♀ [UMSP], M. Sabourin leg.; **Crawford Co.**: Barnum (43.218, -90.839): 1 ♀ [UMSP], 2 Aug 1922, A.M. Holmquist leg.; **Dane Co.**: Madison (43.094, -89.321): 1 ♀ [OSUC], 25 Jun 1916; **La Crosse Co.**: [MASR]; **Oconto Co.**: Lakewood (45.3, -88.523): 1 ♀ [UMSP], 15 Jul 1948, H.E. Milliron leg.; **Polk Co.**: Tewksbury SACN (45.3031, -92.7312): 1 ♀ [CNBL], 25 May 2017, K. Friedrich leg., vac, *Barbareavulgaris*; 2 ♀ [CNBL], 8 Jun 2017, K. Friedrich leg., vac, *Ru.* Sp.; 1 ♀ [CNBL], 4 Jun 2018, K. Friedrich leg., vac, *Ru.* Sp. **Canada: Ontario: Middlesex Co.**: London: [MASR].

### ﻿Other US *Augochloropsis* species

#### ﻿ Augochloropsis (Paraugochloropsis) anonyma

Taxon classificationAnimaliaHymenopteraHalictidae

Cockerell

BB67D262-7F85-5D6A-9BB1-DF7C093E0DAE

Augochlora anonyma Cockerell, 1922: 15 ♀. **Holotype**: ♀ USA, Florida, No Name Key [USNM, catalog #53678 barcode #: 00536758, Type #: 2489]. Images examined by ZP and MA. Online record: http://n2t.net/ark:/65665/347b15a43-e8d1-4195-8eaf-f8ac9cbbec94 (labels read “No Name / Key 3.98 Fla // GN Collins / Collector // CL Pollard / Collector // TypeNo. / 24890 / U.S.N.M. [red label] // Augochlora / anonyma / Ckll. TYPE.”).
Augochloropsis cuprea (in Sandhouse 1937 [in part]: key).
Augochloropsis (Paraugochloropsis) anonyma (in Mitchell 1960: key, redescription of female, first description of male; Hurd 1979: catalog).

##### Diagnosis.

Both sexes of *Augochloropsisanonyma* can be recognized by the short propodeal triangle, which is impressed and narrower than the metanotum (Fig. 17E). *Augochloropsisanonyma* are most likely to be confused with *Augochloropsisviridula*, as they overlap in range, and both have shining integument and poorly developed apical hair fringes on the metasoma. *Augochloropsisanonyma* females can be recognized by the presence of dark pubescence on T2–T4, whereas *Augochloropsisviridula* lacks dark pubescence. In addition, *Augochloropsisanonyma* has the propodeum dorsal surface impressed, shining, and relatively narrow with its medial length slightly less than the medial length of the metanotum (Fig. 17E). In comparison, *Augochloropsisviridula* has the propodeum dorsal surface flat throughout, not impressed, and relatively broad, its medial length dorsally as long (or longer) than the medial length of the metanotum (Fig. 17C).

*Augochloropsisanonyma* is also similar to *Augochloropsisfulgida* because both share the character of dark hairs on the metasoma (see Fig. 23C, D for *fulgida*). However, the narrow propodeal triangle of *Augochloropsisanonyma* (Fig. 17E) separates these two species, as *Augochloropsisfulgida* has the propodeal triangle broader (visible in Fig. 23C).

##### Comments.

*Augochloropsisanonyma* is known from the far southeastern US, and we have examined material from Florida and Georgia (Fig. 18). Mitchell (1960) reports it occurring as far north as North Carolina.

#### ﻿ Augochloropsis (Paraugochloropsis) cuprea

Taxon classificationAnimaliaHymenopteraHalictidae

(Smith) stat. nov.

0562824D-D2EC-5427-97DB-7290F59096AE

**Figure 22.**
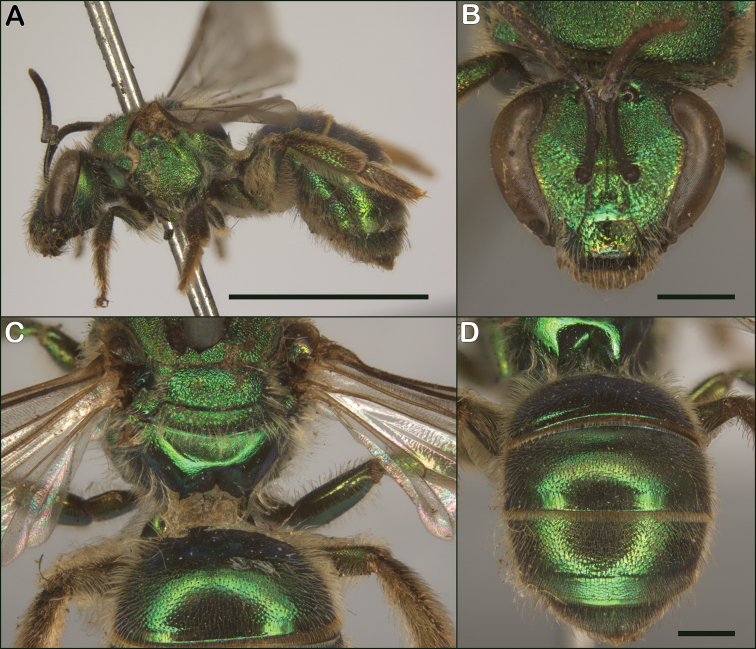
*Augochloropsiscuprea* holotype female **A** lateral view **B** head **C** rear dorsal view of propodeum **D** metasoma. Scale bars: 5 mm (**A**); 1 mm (**B**); 1 mm (**D**). Images provided by Dr. James Hogan (OUMNH).

Augochlora cuprea Smith, 1853: 79 ♀. Images examined by ZP and MA (Fig. 22). **Holotype**: ♀ North America [OUMNH]. (Labels read: “[small square with illegible markings] // HOLOTYPE / Augochloropsis /cuprea (Sm) / J.S. Moure 1957 // Probably the Holotype as labelled. No specimen labelled Type in B.M. / C.D. Michener in litt. 13 VIII 1965”).
Augochloropsis cuprea (in Sandhouse 1937 [in part]: key).

##### Comments.

The type of *Augochloropsiscuprea* (Fig. 22) was located in the Oxford Museum, and based on the label, it was examined by J.S. Moure in 1957 and C.D. Michener in 1965. The specimen is not clearly labeled as the type, but both Moure and Michener agreed that it was likely the holotype. Sandhouse (1937) appears to have only examined the type by proxy through sawfly taxonomist R. B. Benson and Mitchell (1960) did not examine it.

*Augochloropsiscuprea* was considered a junior synonym of *Augochloropsismetallicametallica* by both Moure (1960) and Mitchell (1960). However, examination of the type of *Augochloropsiscuprea* reveals that it is distinct from *Augochloropsismetallica* based on the short T2 fringe (Fig. 22D), the relatively short T3 impressed area (Fig. 22D), the presence of some black hairs on the metasoma (Fig. 22A, D), the more shining propodeum (Fig. 22C), and the short posterior carina of the propodeum (Fig. 22C, D). As a result, we recognize the two forms as heterospecific and thus *Augochloropsiscuprea* as a valid species.

The range of *Augochloropsiscuprea* is unclear as Smith (1853) reports the type locality as “North America”. We have in our possession a single female from Oklahoma (from Four Canyon Preserve headquarters, Ellis County) that may be a match for *Augochloropsiscuprea*, but this must be considered tentative, especially since the Oklahoma specimen lacks the number of black hairs on the metasoma seen in the type. There is also the possibility that *Augochloropsiscuprea* is a Mexican species, especially since multiple Mexican species share the character of the broadly shiny propodeal triangle. The male remains unknown and additional investigation, with more material, is sorely needed.

#### ﻿ Augochloropsis (Paraugochloropsis) fulgida

Taxon classificationAnimaliaHymenopteraHalictidae

(Smith) stat. nov.

978E8C30-228E-50E9-8C5D-A504A558E484

**Figure 23.**
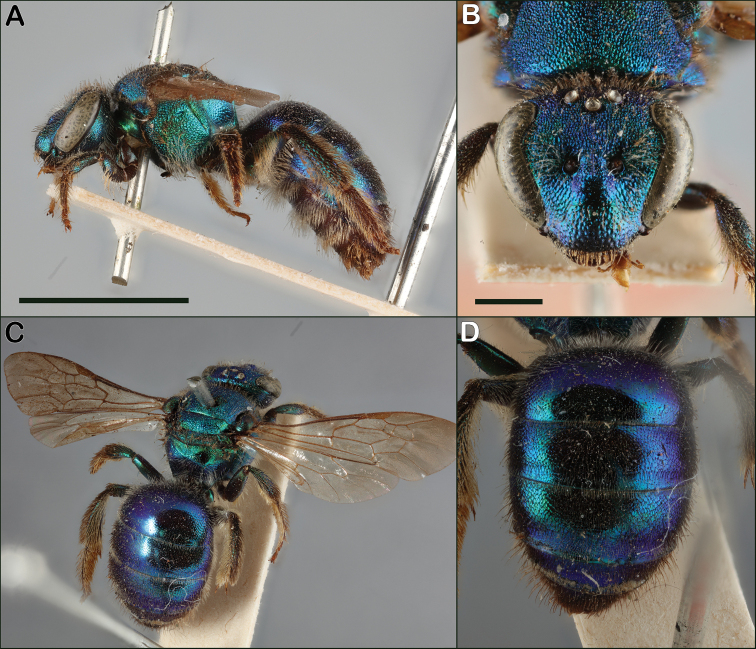
*Augochloropsisfulgida* holotype female **A** lateral view **B** head **C** rear dorsal view **D** metasoma. Scale bars: 5 mm (**A**); 1 mm (**B**). Images provided by Dr. Joseph Monks (NHMUK).

Augochlora fulgida Smith, 1853: 79 ♀. **Holotype**: ♀ USA, Florida, St. John’s Bluff, East Florida [NHMUK014024970]. Images examined by ZP and MA (Fig. 23). Online record: https://data.nhm.ac.uk/object/f5102905-1ee6-44fe-81f5-df87a97b4033 (Labels read “Type / H.T. [circle with red border] // B.M. TYPE / HYM / 14.a.1231 // B.M. TYPE / HYM / augochlora / fulgida / Smith 1853 // fulgida / Type Sm. // E. Doubleday / St. John’s Bluff, / E. Florida. // NHMUK 014024970 [label with QR code]”).
Augochlora fulgida (in Cockerell 1905: taxonomy).
Augochloropsis cuprea (in Sandhouse 1937 [in part]: key).

##### Comments.

We define *Augochloropsisfulgida* differently than previous workers because examination of the type specimen (Fig. 23) revealed that it does not match the species concept used for *Augochloropsismetallicafulgida* by Mitchell (1960). Even though Mitchell (1960) examined the type, it does not key out correctly in his key or match his description. As it stands, the type of *Augochloropsisfulgida* does not match any *Augochloropsis* species we are familiar with. The type female, from St. John’s Bluff Florida (Fig. 18), is most similar to *Augochloropsisanonyma* in that it has erect dark hairs on the metasoma (Fig. 23C, D), but it differs in having a larger and more tessellate propodeal triangle (Fig. 23C) compared to the narrow and shining propodeal triangle in *Augochloropsisanonyma* (Fig. 17E), and the hair fringe on T2 of *Augochloropsisfulgida* appears to be slightly more prominent than in *Augochloropsisanonyma*.

More work is needed to clarify this species as it is currently only known from the type and the male is unknown. However, we have not performed a dedicated search for more material that could match *Augochloropsisfulgida*. It is also a possibility that the type of *Augochloropsisfulgida* is mislabeled and not from Florida or even the United States. However, a more likely explanation is that any *Augochloropsisfulgida* material has been misidentified as *Augochloropsisanonyma* due to the presence of black pubescence on the metasoma.

#### ﻿ Augochloropsis (Paraugochloropsis) sumptuosa

Taxon classificationAnimaliaHymenopteraHalictidae

(Smith)

A9E7120B-EDC0-533B-B6E7-A20A3FCE01D6

Augochlora sumptuosa Smith, 1853: 82 ♀. **Syntype(s?)**: ♀ North America. Type or types missing and presumed lost.
Augochlora lacustris Cockerell, 1922: 14 ♀ (syn. Sandhouse 1937). **Holotype**: ♀ USA, Florida, Lakeland [USNM Type no. 24888]. Images examined by ZP and MA. Online record: http://n2t.net/ark:/65665/32a505d56-7e7d-4fea-ba47-574f3858121f (labels read: “Lakeland, Fla / Nov. 8 1911 // [red label] TypeNo. / 24888 / U.S.N.M. // Augochlora / lacustris / Ckll. TYPE // [yellow label with barcode] USNM ENT 00536780”).
Augochlora floridica Cockerell, 1922: 14 ♂ (syn. Sandhouse 1937). **Holotype**: ♂ USA, Florida, Monticello [USNM Type no. 24889]. Images examined by ZP and MA. Online record: http://n2t.net/ark:/65665/3053e0fc2-b95d-49b6-9630-b55645b3e89d (labels read: “MonticelloFla / Oct. 4–8, 1914 // [red label] Type No. / 24889 / U.S.N.M // Augochlora / floridica Ckll / TYPE. // [yellow label with barcode] USNM ENT / 00536777”).
Augochlora sumptuosa (in Robertson 1887: floral record).
Augochlora humeralis (in Smith 1910: biology).
Augochloropsis caerulea (in Sandhouse 1937 [in part]: key).
Augochloropsis (Paraugochloropsis) sumptuosa (in Mitchell 1960 [in part]: key, redescription; Hurd 1979 [in part]: catalog).

##### Diagnosis.

*Augochloropsissumptuosa* is most similar to *Augochloropsishumeralis* (refer to the diagnosis of that species to separate them).

##### Comments.

We use *Augochloropsissumptuosa* in a more restricted sense than previous authors because we have split it into two species: *Augochloropsissumptuosa* and *Augochloropsishumeralis*. Now, *Augochloropsissumptuosa* refers to the species occurring in the southeastern United States though the exact range is unknown at this time, and it remains to be seen to what degree, if any, the range of the two species overlaps.

The type or types of *Augochloropsissumptuosa* have been lost. The type could not be located by Sandhouse (1937) or Mitchell (1960). The last report of possible types is from Cockerell (1897), who stated that “Col. C.T. Bingham” examined syntypes in the Natural History Museum (London, UK). However, there are currently no specimens in the Natural History Museum that could possibly be syntypes (J. Monks, pers. comm., Jun 2021). Despite the missing types, it is our opinion that there is not a need for a neotype because the identity of *Augochloropsissumptuosa* can be determined from the original description. Specifically, the original description states “the base of the metathorax enclosed by an arched ridge, the enclosed space granulated, the sides of the truncation margined by sharp carinae.” This description matches the southeastern species (which has the propodeal triangle surrounded by a weak semicircular carina; Fig. 17D) but not the species found in the Midwest. On this basis, we are retaining the name *Augochloropsissumptuosa* Smith for the southeastern species. The oldest available name for the midwestern species is *Augochloropsishumeralis* (Patton), the types of which were collected in western Kansas.

More work remains to be done on the taxonomy of *Augochloropsissumptuosa* because we have not critically evaluated the status of two synonyms: *Augochloralacustris* Cockerell and *Augochlorafloridica* Cockerell. They were originally synonymized with *Augochloropsissumptuosa* by Sandhouse (1937), and Mitchell (1960) agreed with that designation, stating “Examination of the types has failed to reveal any significant difference that would justify the recognition of either *lacustris* or *floridica*.” We have examined images of the types, which are clear enough for us to tentatively agree. However, given that we have split *Augochloropsissumptuosa* into two species and there is potentially a third similar species in Florida, these types should be critically reexamined as part of a reevaluation of the Florida fauna.

### ﻿Additional unknown *Augochloropsis* species in the United States

We are aware of at least four additional potential species of *Augochloropsis* in the United States. We are listing them here in order to alert readers to their presence, as many have been incorrectly lumped together under existing species, particularly *Augochloropsismetallica*. However, we do not treat them further. We lack sufficient material of these species, and it is unknown whether they are undescribed or not, as they may be described from Mexico or they may be one of the many poorly known species described by Cockerell. The potential species and their locations include:

- Arizona: A species with a broad and shining propodeum in the female (M. Arduser, unpublished).
- Florida: A species similar to *Augochloropsissumptuosa* seen in material from Archbold Biological Station (M. Arduser, unpublished).
- Texas: A species that has a unique propodeal triangle (Fig. 17F) and an intermediate T2 comb (Fig. 15C) that falls between *Augochloropsismetallica* and *Augochloropsisviridula* (Z. Portman, unpublished; from material in UMSP and OSUC). This species may have contributed to the confusion by previous authors who believed that *Augochloropsismetallica* and *Augochloropsisviridula* were a single variable species.
- Texas: A species similar to *Augochloropsishumeralis* (M. Arduser, unpublished).

## ﻿Conclusions

Here, we have revised the *Augochloropsis* of the Midwest and made additional changes to the *Augochloropsis* of the broader United States. This work will allow for the confident identification of the species in the midwestern United States and allow the species’ ranges to be better understood. However, there are areas of the southern United States (particularly Florida and Texas) where any *Augochloropsis* identifications must be undertaken with great care due to the number of undescribed or unknown species. We estimate there are an additional four species of *Augochloropsis* in the United States that are unknown or undescribed, not counting *Augochloropsisfulgida*, which is only known from the type and has the male now unknown. In addition, more work needs to be done to check the status of some of the current synonyms of *Augochloropsissumptuosa* and *Augochloropsishumeralis* from Texas and Florida. Even the genus name may change at some point, as Gonçalves et al. (2022) advocate for raising the subgenus Paraugochloropsis to genus level.

The taxonomic changes and identification resources provided here will allow for more accurate identification of *Augochloropsis* and the other shiny green Halictinae. However, similar to the situation in *Augochloropsis*, more taxonomic work is still needed in the other shiny green Halictinae. For example the *Agapostemon* of the United States were last revised 50 years ago (Roberts 1972; Janjic and Packer 2003; Sheffield et al. 2021), the *Augochlora* of the United States have never been revised, and the molecular and morphological diversity found in *Augochlorellaaurata* suggests it is potentially a cryptic species complex (Ordway 1966; Sheffield et al. 2009). Given the identification issues surrounding what were the former *Augochloropsismetallica* subspecies, it is especially important that researchers cite the taxonomic concepts and identification resources they use and save voucher specimens (see Packer et al. 2018). In addition, we recommend that non-peer-reviewed identification resources should be avoided whenever possible, as they often have errors and lack a version of record. Indeed, many non-peer-reviewed works would not pass peer review, and the widespread use of these error-ridden and out-of-date identification resources (particularly the keys on discoverlife.org) are contributing to the high rates of misidentifications in bees.

Our work also demonstrates the difficulty, indeed the futility, of attempting to monitor many bee groups that are in taxonomic disarray (Portman and Tepedino 2021; Tepedino and Portman 2021). Here, we have altered the species concept of essentially every *Augochloropsis* species in the United States and split what was formerly *Augochloropsismetallica* into five species (Table 1). This will necessitate that the majority of existing identifications be checked and updated, which is impossible for monitoring schemes or other studies that do not preserve their specimens (Packer et al. 2018), and it demonstrates one of the major issues with digitizing old museum specimens without first updating them to modern taxonomic concepts. While the taxonomic changes made here will no doubt cause headaches as specimens are checked and names updated, this is a predictable consequence of a genus going 60+ years without a revision. The taxonomic issues seen in *Augochloropsis* are not an isolated problem, as demonstrated by the high rate of new species described in recent revisions of the North American bee fauna (e.g., 15 new species of *Epeolus* (Onuferko 2018); 20 new species of “red-tailed” *Lasioglossum* (Gardner and Gibbs 2020)). This high rate of new species discovery and taxonomic changes will continue in bee genera and subgenera that either lack revisions or have not been revised in the last 50 years (e.g., *Melissodes*, *Nomada*, *Sphecodes*, many *Andrena*, etc.), particularly since prior taxonomic research on those groups predates molecular tools and high-resolution images.

**Table 1.** Comparison of names and species concepts applied over various keys and revisions of *Augochloropsis*. Dashes indicate that the species was not treated by the author.

Robertson 1902	Sandhouse 1937	Dreisbach 1945	Mitchell 1960	Current name
*fervida* (Smith)	*cuprea* (Smith)	*cuprea* (Smith)	*metallicametallica* (Fabricius)	*metallica* (Fabricius)
–		–		*cuprea* (Smith)
–		–		*fulvofimbriata* (Friese)
*viridula* (Smith)		*viridula* (Smith)	*metallicafulgida* (Smith)	*viridula* (Smith)
–		–		*fulgida* (Smith)
–		–	*anonyma* (Cockerell)	*anonyma* (Cockerell)
–	*caerulea* (Ashmead)	*humeralis* (Patton)	*sumptuosa* (Smith)	*humeralis* (Patton)
–		–		*sumptuosa* (Smith)

## Supplementary Material

XML Treatment for Agapostemon

XML Treatment for Agapostemon (Agapostemon) angelicus

XML Treatment for Agapostemon (Agapostemon) melliventris

XML Treatment for Agapostemon (Agapostemon) sericeus

XML Treatment for Agapostemon (Agapostemon) splendens

XML Treatment for Agapostemon (Agapostemon) texanus

XML Treatment for Agapostemon (Agapostemon) virescens

XML Treatment for Augochlorella

XML Treatment for Augochlorella aurata

XML Treatment for Augochlorella persimilis

XML Treatment for Augochlora

XML Treatment for Augochlora (Augochlora) pura

XML Treatment for Augochloropsis

XML Treatment for Augochloropsis (Paraugochloropsis) humeralis

XML Treatment for Augochloropsis (Paraugochloropsis) metallica

XML Treatment for Augochloropsis (Paraugochloropsis) viridula

XML Treatment for Augochloropsis (Paraugochloropsis) anonyma

XML Treatment for Augochloropsis (Paraugochloropsis) cuprea

XML Treatment for Augochloropsis (Paraugochloropsis) fulgida

XML Treatment for Augochloropsis (Paraugochloropsis) sumptuosa

## Footnotes

Addendum - While this article was in press, a male Agapostemon angelicus was identified by ZP from southwestern Minnesota. It was collected in 2021 in Jackson County. This represents the first record of Agapostemon angelicus from Minnesota.
